# Preliminary Review of the Diploid Taxa in *Hieracium s*.*s*.

**DOI:** 10.3390/plants14071057

**Published:** 2025-03-29

**Authors:** Giacomo Baldesi, Jean-Marc Tison, Simone Orsenigo

**Affiliations:** 1 Department of Earth and Environmental Sciences, University of Pavia, Via Sant’ Epifanio 14, 27100 Pavia, Italy; simone.orsenigo@unipv.it; 2 Independent Researcher, Chemin du Valentier, F-38540 Heyrieux, France; jmltison@gmail.com

**Keywords:** Asteraceae, Cichorieae, *Hieracium*

## Abstract

A review of the known diploid species in *Hieracium* L. *s.s.* (Asteraceae, Cichorieae) is presented. This article aims to summarize the current knowledge of the taxa with the basic ploidy level in the genus (2*n* = 2x = 18), as these entities are supposed to have originated the outstanding diversity observed nowadays, which is largely ascribable to polyploid microspecies. The study of extant diploid species is crucial for the understanding of the speciation dynamics that occurred in hawkweeds. All available cytological data in the literature, pertinent to Europe and adjacent countries, are included to have an overview of the obligately sexual species in this genus and highlight gaps and uncertainties. In order to preliminarily investigate the geographical distribution, all records are georeferenced and projected on a map to highlight the hotspots of *Hieracium* diversity. A brief account of each taxon is included, with some additional considerations and remarks on doubtful records.

## 1. Introduction

*Hieracium* L. *s*.*s*. (or *Hieracium* subg. *Hieracium*) is among the most diverse, hence most complicated, and species-rich genera in Europe and possibly worldwide. It comprises perennial herbs mainly found in temperate regions of Europe, Asia, and North America. Hawkweeds have adapted to the most varied habitats, including forests, forest margins, various grasslands, and rocky outcrops from sea level to well above the tree (timber) line. The impressive degree of morphological variability is challenging and poses serious problems for species delimitation; this subject is still a matter of debate [1,2]. Genus-specific concepts of species exist [1,3,4]; resulting in genus-wise, regionally different classifications which have complicated the study of the genus as a whole at the European level (see the Central European vs. the Scandinavian approach) [1,2,4,5,6]. Traditionally [7,8,9], the genus has been split into sections, or rather groups of species sharing morphological similarities and putative common ancestry, which have been reviewed afterward [10]. Depending on the taxonomic treatment, the number of species can vary from 500 to 5000 [1,4,5,6]. Moreover, the broad spectrum of morphological characters often lacks clear discontinuities, hampering taxa identification and discrimination. Consequently, *Hieracium s*.*s*. has a reputation for being a “taxonomist’s nightmare” and unfortunately it is seldom collected by botanists, reducing available specimens and sampling coverage.

Hybridization along with introgression, and subsequent polyploidization tightly coupled with the switch to apomixis [11] are considered the key factors in the radiation of *Hieracium*, originating (resulting in) the great diversity that we observe nowadays [12,13,14,15]. This extreme variation might be partly due to other processes that are still largely unknown, such as “pseudo-sexual” recombination among the different chromosome copies and structural or point mutations involving genes associated with morphological characters [6]. The genus is to be regarded as a complex of species largely dominated by polyploids (mainly triploids and tetraploids), which for the most part are obligate apomictics [16,17,18,19,20]. Apomictic species notably account for all the northerly taxa (except for one single widespread diploid species) [20]. *Hieracium* is considered a model system for the study of apomixis; development of the unreduced embryo sac conforms to the “*Antennaria* type” of diplospory, where meiosis is fully omitted [21,22,23], and embryo development starts precociously, even before anthesis [24,25]. Maternal transmission of cpDNA was confirmed in the genus [12]. Occasional anomalies or residual sexual processes in the development of the female gametophyte were reported in the literature [24,25]. In fact, facultative apomixis was postulated by different authors [7,8,9,12,24]. Sporadic meiosis and tetrad formation were observed in a few apomictic species [22]. Additionally, rare sexual events were detected in just a few individuals appearing to be linked to determinate genotypes; precocious embryony might not be so stringent and such facultatively apomictic lineages are potentially able to reproduce sexually at least occasionally [23]. On the other hand, polyploids are able to produce pollen via meiosis in varying proportions: male fertility spans from complete sterility to normal pollen fertility [25,26,27,28,29,30]. Considering the depicted scenario, interactions among different species, especially with different ploidy levels, need to be carefully investigated as reproductive strategies and population dynamics are yet to be fully understood.

## 2. Phylogeny and Evolutionary Scenarios

*Hieraciinae* Cass. ex Dumort includes, other than *Hieracium*, the closely related genera: *Andryala* L., *Hispidella* Barnadez ex Lam. and *Pilosella* Hill [31] (regarding *Schlagintweitia* Griseb. see considerations on *Hieracium intybaceum* All.); this subtribe has a rather complex evolutionary history resulting from ancient intergeneric hybridization [32,33]. Phylogeny reconstruction of the genus *Hieracium* has proved to be quite challenging, taking into account the combination of extensive hybridization, apomixis, and polyploidization; the lack of divergence of various molecular markers prevented the resolution of most interspecific relationships [15,33,34,35,36]. Moreover, chromosomal patterns were suspected to be not reliable to infer species relationships in *Hieracium* due to the dynamic organization of rDNA loci [33]. To date, two major clades were detected by a single nuclear marker (ETS) suggesting a basal split (“Eastern” and “Western” clades), further corroborated by differences in DNA content [15,34,35,37]; additionally, major cpDNA haplogroups were retrieved. Extinct or possibly unsampled lineages together with reticulation and incomplete lineage sorting are possibly the main factors limiting resolution in molecular analyses [15,34,35]. Inference of phylogenetic relationships in highly reticulate and mostly polyploid groups is still a considerable challenge. Around half of the taxa investigated in phylogenetic analyses are suspected to be of hybrid origin [15], independently of ploidy level, and including numerous diploids. The hybrid origin of taxa can be easily overlooked or misinterpreted if extant species diversity is not representative of the “original/whole” variability; extinct ancestral lineages may limit the inferral of parental species’ contribution and their distinctive characters. Intricate past interspecific hybridization appears evident both morphologically [7,8,9] and at the molecular level [15,34,35], yet reticulation of characters limits the understanding of its precise dynamics.

Considering the absence of apomixis in diploids [19,23,28], the emergence of primordial agamospecies is linked to interspecific hybrids that acted either as donors of pollen or parents of offspring, both variable in ploidy level [13,15,38]. The coexistence of sexual diploids and polyploids, producing potentially fertile pollen, triggered recurrent hybridization and the emergence of different apomictic hybrid swarms capable of occasional backcrossing with the diploid parents. First-generation hybrids often show fertility problems [12,13,14,39], and generally backcrossing with the parental taxa is reputed necessary for the stabilization of polyploid/apomictic lineages [40].

In summary, the most likely evolutionary scenario implies that “few” diploid species survived the glaciations and experienced population bottlenecks. These ancestral species underwent speciation, possibly still as diploids, when recently deglaciated habitats became available; subsequent speciation occurred rapidly and with little divergence according to available molecular data [15,33,34,35,36,38]. Secondary contact [41] resulted in hybridization between isolated species groups; the crossing of lineages differing in genome size [15] is supposed to have altered regular meiosis inducing the rise of polyploids and subsequently apomicts. These newly fixed agamic lineages rapidly spread and colonized available habitats more efficiently compared to their diploid parents [19,42]. This peculiar population structure possibly results from the interactions of reduced gene flow (except for diploids), considering the limited pollen viability in some species [23,29,30], mentor effect (self-fertilization resulting after a breakdown in self-incompatibility) [28], and precocious embryony [22]. This model of cytotypes’ distribution follows the concept of geographical parthenogenesis where conspecific or closely related sexual and asexual organisms have different distribution patterns [19,34,40].

## 3. Considerations on Diploid Taxa

Diploid *Hieracium* diversity is reported as particularly high in the European mountains (Alps, Pyrenees, Carpathians, Balkan Peninsula) and in Western Asia. With a base number of x = 9 (2*n* = 18), diploids are considered rare and thought to be confined mainly to Southern or Eastern European glacial refugia (e.g., Alps, Carpathians, Pyrenees) [4,17,18,20,23,35,43,44,45,46,47,48,49,50,51]. These species have been proven obligately sexual [19,23,28], and seed formation follows fertilization of the haploid egg cell and the diploid central nucleus by a haploid spermatic cell [24,52,53]. Strict sexuality and sporophytic self-incompatibility (SI) were confirmed in diploids by isolation experiments [28,54]. However, heterospecific pollen can trigger the failure of the self-incompatibility system inducing self-pollination (mentor effect) [14,28,55]. Surprisingly, little or no information is available about general physiological traits including seed production, dispersal, and germination. Only a single research paper [56] investigates the differences between sexual and apomictic cytotypes of the same taxon in terms of seed production and dispersal. Advanced karyotype analyses are limited to only a few diploid species: *H. alpinum* L. [38,57], *H. bracteolatum* Sm. [58], *H. intybaceum*, *H. prenanthoides* Vill. [36,59], *H. transylvanicum* Heuff. [60], and *H. vranceae* Mráz [35]; karyotyping may prove to be a useful tool to investigate relationships in this genus and detect hybridization phenomena.

At present, interspecific hybridization among diploids is considered rare and it has been only occasionally detected in nature [35,39,43] or artificially produced [13,14,28,55]. The coexistence of such species is infrequent, often having restricted and allopatric ranges with little or no overlap, and the mentoring effect acts as a strong barrier. A single event of spontaneous polyploidization following the interspecific crossing of diploids has been recorded so far [13]. In another case, hybridization between diploids as seed parents and a polyploid apomictic species was considered [12]. As only recently demonstrated [36], the same diploid parental species can potentially give rise to different phenotypically distinct hybridogenous offspring, depending on the contribution of each taxon and its gametes. Additionally, the relative abundance of a given taxon in determinate sites is correlated to the direction of the hybridization together with habitat characteristics and ecophysiological traits of the species involved [13]. It must be noted that a few species were proven to occur in mixed ploidy populations (di- and triploids) and/or both diploid and triploid cytotypes are recorded from the species in its range, e.g., [15,30,61].

In abstracto, the “sexuals-first” concept [62] should be the preferential way to approach the genus *Hieracium s.s*. The understanding of the origin, evolution, and phylogenetic relationships of species complexes is subordinate to the delimitation of sexual progenitor species [35,62]. In fact, phylogenetic analyses, including both diploid and polyploid *Hieracium* taxa [15,34,35] suggested that diploids display the whole genetic diversity within the genus, while polyploids are the result of various combinations of different diploid lineages. However, not all diploid species and only very few of the extremely numerous polyploid species have been included in these studies.

Diploid taxa are preferably broadly circumscribed so that their natural range of variation, resulting from the interaction of gene flow and intraspecific cohesion, is better represented [35]. Natural variation, which is far greater than usually recognized, should be emphasized and thoroughly evaluated. A wide morphological concept seems to be the most appropriate at present as little is known about potential phenotypic plasticity and the genetics underlying it. In this regard, experimental taxonomy, in particular crossing experiments and cultivation [14,55], and subsequent observation over different growing seasons (ideally both in and ex situ when feasible), shall be a very useful tool to integrate morphological and genomic data.

Up to 2019, ca. 25 diploid species were reported in this genus [17,23]. Four of the known diploid species have been recently described [35,45,46,49], and most of the others were described a long time ago while their ploidy was investigated much later.

## 4. Ploidy Analyses/Data

Determination of ploidy levels is of high priority to evaluate the mode of reproduction [23]. Chromosome counting has been the traditional method to determine ploidy. In recent years, the use of flow cytometry has allowed the screening of many samples by estimating genome size, although for confirmation of the genome data traditional chromosome counting is still highly recommended. This tool appears extremely useful as well for investigating reproductive pathways and polyploidization events if employed for seed screening [19,23].

Many chromosome counts for different *Hieracium* species have been published so far, especially in the latest years, e.g., [63,64,65,66,67], though there are still considerable gaps in our knowledge of ploidy levels throughout the range of the genus. This appears particularly severe in Southern Europe, for instance Italy and France, etc., where more diploids are likely to be detected. This knowledge is crucial for the understanding of the evolutionary processes in the genus and for making taxonomic considerations accordingly.

This review will consider mostly publications dealing with diploid species, as polyploid counts greatly outnumber diploid ones. All published cytological data (mainly chromosome counts), pertinent to Europe and adjacent countries, are included to have an overview of the known diploid species in this genus. 

Available publications and databases [63,64,65,66,67] have been searched and records of diploid *Hieracium* species and their locations are reported in the dataset (accessible in Supplementary Material S1). Records provided only with a generic locality are approximated.

## 5. Species List

Species are presented in alphabetical order and for each, a brief overview is given, in which distribution and habitat are indicated together with some considerations on their morphology, taxonomic status, and possible relationships and affinities with other species. Taxa are referred to by their specific name, often corresponding to their basionyms. If such a combination is not available, the subspecific name is reported. Considering the non-negligible degree of taxonomic uncertainty, some of the mentioned species need further in-depth studies to confirm their identity and specificity.

- ***H. alpinum*** L.

The native range of this species is arctic and alpine Europe to W. Siberia, Central Asia, and Greenland [68,69]. In Central Europe it is found in the Alps, the Sudeten Mts., and eastwards to the Carpathians. Some isolated populations are reported from the Vosges (France), the Harz Mountains (Germany), and the Vranica Plateau (Bosnia and Herzegovina) [54]. It typically grows in non-calcareous arctic tundra habitats and in the subalpine or subarctic biome in open-canopy grasslands on mountain summits and highest slopes where it can be locally widespread [54]. The diploid cytotype is known only from the Eastern and Southern Carpathians (Romania and Ukraine) and numerous counts are recorded [12,28,30,50,54,70,71]. Triploid cytotypes are also reported in the literature [63] and are distributed in the remaining range of the species. The apomictic and sexual cytotypes of *H*. *alpinum* are geographically isolated [19,54]. This species is placed in sect. *Alpina* (Griseb.) Gremli has a distinctive morphology and habit: usually with unbranched stems bearing one single capitulum (scapose habit) or occasionally each accessory rosette may produce a single stem. *Hieracium augusti-bayeri* (Zlatník) Chrtek f., differing only by being glabrous, is included in the range of variability of *H. alpinum* and represents a morph of this sexual taxon [35]. Diploid hybrid plants between sexual diploid cytotypes of *H*. *alpinum* and *H. umbellatum* L., corresponding to *H. grofae* Woł., were found in the Ukrainian Eastern Carpathians [39]; additionally, the sterile diploid hybrid *H. krasanii* Woł. results from the crossing of *H. transylvanicum* and *H. alpinum* 10]. These occasional hybrid species are included within *H. alpinum* in the present list. Meiotic tetrads, which were subsequently aborted, are reported from *H. alpinum* [25]. Phylogenetically, this species formed a lineage of its own included in the “Eastern” clade and its cpDNA haplotype appears unique (found only in hybrid taxa directly linked to the species) [15,34,35].

- ***H*. *bifidum*** Kit. ex Hornem. *s*.*l*.

This is a Central and North European species [68]. It is a predominately mountainous species but with ample altitudinal tolerance and a clear calciphilous tendence [69]. Placed in sect. *Bifida* T.Tyler, it shares morphological similarities with *H. murorum* L. *s*.*l*. and/or *H. stelligerum* Froel. Only a single diploid record is present in the literature [26], and the analyzed plants are of unknown origin and cultivated. Merxmüller [43] cites this only count and postulates that the Rosenberg’s plant corresponds to *H. bifidum* subsp. *canitiosum* (Dahlst.) Zahn, which has subsequently been reported to be triploid [20]. In all other counts [20,63], this species resulted in polyploid. *Hieracium bifidum s*.*l*. is most likely a very complex group of species and more studies, especially in the Alps and Prealps, are needed. Considering the impressive degree of variability reflected by the numerous taxa included within it [7,8,9], it is likely that diploid lineages exist in the group of “bifidoid” species. The triploid accession included in molecular analyses was placed in the “Western” clade according to nuclear and cpDNA haplotypes [15,34,35].

- ***H. bracteolatum*** Sm.

This Circum-Aegean relict species occurs from North Macedonia to Greece [51,68], typically growing in deciduous forests [7,8,9]. It is morphologically unique, deserving its own monotypic section (sect. *Bracteolata* Zahn), having long spicate inflorescence with subsessile capitula. Diploid counts are known only from the Greek Islands of Evia and Thasos [51,58], while triploid [37] and tetraploid [72] counts were also reported in the literature. This plant appears similar to *H. scamandris* Zahn from Western Turkey [68] with unknown ploidy levels [51]. Molecular analyses retrieved the plant as an interclade hybrid, presenting both “Western” and “Eastern” sequence variants, but variation was too low to separate it from other taxa of the same subclade [15,34,35].

- ***H. cerinthoides*** L*. s.l.*

This group of species is native to France and Spain, where it is found in the Pyrenees and the Cantabrian range growing in various rocky habitats, between 600 and 2400 m a.s.l. [73,74]. A single diploid record is reported [75], and the identity of the counted plant is dubious as it could belong to another taxon of the *H. cerinthoides* group [18,76] or sect. *Cerinthoidea* Monnier. *Hieracium cerinthoides, H. gymnocerinthe* Arv.-Touv. & Gaut. (≡*H. cerinthoides* subsp. *gymnocerinthe* (Arv.-Touv. & Gaut.) Zahn), and *H. ramondii* Griseb. share some strong morphological (absence of eriopodous base and glabrescent leaves) and ecological similarities (rupicolous species); they are distinguished only by the involucre indumentum: mostly glandular in *H. gymnocerinthe*, mostly hairy in *H. ramondii* and intermediate in *H. cerinthoides* [73]. The name has been recently typified [77] and corresponds to the apomict morph. As recognized in Flora Gallica, *H. cerinthoides* series includes seven scarcely distinct taxa comprising also the above-mentioned species (*H. cerinthoides*, *H. chamaecerinthe* Arv.-Touv. & Gaut., *H. fourcadei* de Retz, *H. gymnocerinthe*, *H. mucronatum* Arv.-Touv. & Gaut. (≡*H*. *adenodontum* subsp. *mucronatum* (Arv.-Touv. & Gaut.) Mateo, Egido & Gómiz), *H. ramondii*, *H. trichocerinthe* Arv.-Touv.) [74]. It is likely that three groups of diploids exist in the Pyrenees: *H*. *ramondii* (possibly conspecific with *H*. *obovatum* Lapeyr.) in the West, a taxon ascribable to *H*. *platycerinthe* Arv.-Touv. & Gaut. (distinct from the suspected apomictic *H*. *rhomboidale* Lapeyr.) in the Central part, and *H*. *gymnocerinthe* (possibly conspecific with *H*. *axaticum* Arv.-Touv. & Gaut.) in the East. More accurate investigations are needed to ascertain their relationship. The triploid accession analyzed in the phylogenetic studies (sub *H*. *cerinthoides*) is possibly of hybrid origin (“Western” clade and “Pyrenean” subclade), and its cpDNA haplotypes place it among the Pyrenean species [15,34,35].

- ***H. dollineri*** Sch.Bip. ex Neilr.

This species is native to Central Europe up to Croatia [68]. It is typically associated with rocky and calcareous habitats and is found between 100 and 2200 m a.s.l. [69]. The first and only diploid counts come from Germany [78] on plants identified as the nominal subspecies. This record confirms the existence of diploid lineages in this species, which resulted in otherwise triploid [79]. Traditionally interpreted as the intermediate between *H. glaucum* All. and *H. bifidum* [7,8,9], this species might actually have been involved in the origin of some species grouped in sect. *Drepanoidea* Monnier (possibly in combination with *H. porrifolium* L. or other taxa), from which it was recently segregated to sect. *Dollinera* Gottschl. [69]. Morphologically the plant is distinguished by its clearly petiolate leaves with usually dentate to uncinate margins, the few cauline leaves (compared to members of sect. *Drepanoidea*), the epilose or scarcely pilose peduncles and the eglandular and hairy involucres with abundant stellate hairs. It was not included in any molecular study.

- ***H. eriophorum*** St.-Amans

This plant has a restricted range limited to the Southern Atlantic coast of France in the Aquitaine region, being known only from a narrow stretch of ca. 80 km of the Atlantic sea coast [74,80], possibly occurring in adjacent territories of the Basque country such as the Gipuzkoa coast, although no specimens have been collected from this area [73]. It typically grows on sand and semi-stabilized dunes near the shore [74]. Only diploid counts have been reported for this species [18,43]. Placed in sect. *Eriophora* (Arv.-Touv.) Zahn, it is distinctive in its prostrate habit and dense indumentum of long hairs on both sides of the leaves; the degree of morphological variability includes *H. prostratum* DC. (characterized by the less hairy involucres), previously treated as a separate species, now considered a synonym of this taxon [80]. In the Gipuzkoa region and in the adjacent French territories, the hybrid *H. lavernellei* Timb.-Lagr. (≡*H*. *eriophorum* subsp. *lavernellei* (Timb.-Lagr.) Greuter) is considered the result of the crossing with *H. umbellatum* [73]. *Hieracium eriophorum* is a late flowering species sharing some similarities with *H. umbellatum* and related species. This species likely originated via adaptation to the sand dunes habitat from the widespread *H. umbellatum*. In the last two centuries, this sand dune endemic has apparently experienced a significant contraction of its range due to habitat loss and fragmentation caused by erosion and urbanization [80]. Molecular analyses point out its possible origin as a recent offspring of *H. umbellatum*; cpDNA haplotypes and its placement in “Eastern” clade support this hypothesis [15,34,35].

- ***H. gouanii*** Arv.-Touv.

Found only in NE Spain and S France (Catalonia and the eastern part of the Pyrenees), this species is reported growing on various substrates in habitats including rocky outcrops, slopes, and walls, between 800 and 1900 m a.s.l. [73,74]. Only a single diploid count is reported from the Eastern Pyrenees close to the border between France and Spain [18]. Traditionally treated as a subspecies of *H. cordifolium* Lapeyr. (*H. cordifolium* subsp. *gouani* (Arv.-Touv.) Zahn) [7,8,9,81] and included in sect. *Cerinthoidea*, this species is considered distinct by the robust habit, taller stems, glabrescent, and coriaceous leaves, which are more numerous (with 2-5(-8) cauline leaves) [73,76]. In its range of distribution, the plant seems to hybridize with *H. neocerinthe* Fr. (*H. lagascanum* Arv.-Touv. & Gaut.) [73]. *Hieracium cordatum* Scheele ex Costa is tentatively included under this species as it is regarded as the occasional hybrid *H*. *gouanii* x *H*. *legrandianum* Arv.-Touv. [74] or a distinct taxon showing strong affinities with its putative parental species. It is very likely that the diploid counts reported for *H*. *hispanicum* [43], which is reputed a distinct, apomictic species, are referrable to *H*. *cordatum* instead and this record is tentatively placed under this taxon until further evidence. A triploid count is reported from the type locality of *H*. *cordatum* (sub *H*. *cordifolium*) [47]. The accession of *H*. *gouanii* included in the molecular analyses resulted in an interclade hybrid (“Eastern” and “Western” clade) and its cpDNA haplotypes derived from the Pyrenean species [15,34,35].

- ***H. gymnocephalum*** Griseb. ex Pant.

This species is found only in the subcontinental and Mediterranean mountain ranges of the W Balkan Peninsula (Albania, Greece, ex Yugoslavia) [68]. It typically grows on limestone or calcareous rocks in mountain environments [82]. Diploid counts are reported [37,83]. The whole *H. waldsteinii-gymnocephalum* group needs a revision considering its morphological variability [82,84]. This species belongs to sect. *Pannosa* (Zahn) Zahn, distinguished by the strongly plumose indumentum. Its center of diversity appears to be in the mountains of Montenegro [84,85]. The diploid and triploid accessions included in the molecular studies were revealed to be interclade hybrids with a unique cpDNA haplotype much similar to other closely related taxa (*H. plumulosum* A.Kern.) [15,34,35].

- ***H. intybaceum*** All.

This taxon is distributed all over the Alps with some isolated populations in the Vosges and the Schwarzwald Mountains [69,74,86]. It typically grows on siliceous bedrock in the subalpine and alpine belts, between 800 and 2600 m a.s.l. [69,74,86]. Diploids are distributed across the Alps in Switzerland, Austria, and Italy [18,23,37,86,87,88], while tetraploids seem to be confined to the Western Alps and the Vosges to a rather small geographical area [86]. Placed in sect. *Intybacea* W.D.J.Koch, it is distinct among all *Hieracium* species by its pale sulfury ligules, the constantly yellow stigmas (only in the diploid cytotype), and the abundance of glandular hairs on all vegetative parts [74]. *Hieracium intybaceum* has been retrieved as a sister group to the rest of the subtribe *Hieraciinae* [15,32,34,35] and based on these considerations the name *Schlagintweitia intybacea* (All.) Griseb. was resurrected [89]. It is better to keep this taxon within the genus *Hieracium* until further evidence considering that more recent molecular data revealed high similarities with other species in the genus [38] and suggested its placement among other Western European species [33]. In virtue of its genetic and morphologic distinctiveness from all other species of *Hieracium s.s.*, this species was employed for detecting hybridization phenomena [36]. Contrasting with the trends found in other *Hieracium* species, polyploid lineages appear to be less widespread than the diploid ones, this might indicate the recent origin of the tetraploid cytotype [86].

- ***H. jaubertianum*** Timb.-Lagr. & Loret (≡*H*. *glaucinum* Jord. subsp. *jaubertianum* (Timb.-Lagr. & Loret) O.Bolòs & Vigo)

It is recorded from France, Italy, Spain, and Switzerland [69]. It is typically found in the garrigues habitat [73,74]. Only a single diploid count is reported [90], otherwise, polyploid counts are known [91]. This species was previously included in the numerous subspecies of *H. glaucinum* [7,8,9] (see sect. *Oreadea* (Fr.) Arv.-Touv.). It is distinguished by the usual presence of conspicuous setiform rigid hairs on the leaf margin together with other leaf characters and the involucres densely covered with stellate hairs, more densely at the margins of bracts, often with white simple hairs having a much reduced or absent black basis [74,81]. It is recognized as a distinct species forming its own series (*H. jaubertianum* series) [74]. Additional analyses are needed to delimit its range of distribution and its morphological variability. This species was not included in any molecular analysis.

- ***H. kittanae*** Vladimir.

Endemic to Bulgaria, it is known only from few localities in the Central Rhodope Mountains. This relict species is found on limestone rocks and cliffs in humid sites at middle altitudes [45]. Only diploid counts are recorded [18,37,45]. Considerable morphological variation is reported, especially in the indumentum of peduncles and involucres (glandular and simple hairs in variable proportion). Morphologically, it can be placed in the *H. glaucinum* group [35,45] (see sect. *Oreadea*). This taxon was assessed as Critically Endangered (CR B1ab(iii) + 2ab(iii), according to IUCN criteria, because of its restricted distribution and possible habitat deterioration [45]. According to molecular analyses, it forms a separate lineage within the “Eastern” clade and presents the highest number of polymorphisms; its cpDNA haplotypes are identical to those of *H. pannosum* Boiss. and *H. petrovae* Vladimir. & Szeląg [15,34,35].

- ***H. laniferum*** Cav.

Endemic to the Iberian Mountains and Pyrenees, it grows in crevices on calcareous rocks, between 500 and 1950 m a.s.l. [68,73,81]. Its distribution comprises both sides of the Pyrenees, the Pre-Pyrenees, and the Catalan Pre-Coastal Range up to the Ports de Tortosa-Beseit. Two counts are reported from the Southern Catalonia Mountains [43,47]. It was traditionally treated as a subspecies within *H. ramondii* [7,8,9,81] and is placed in sect. *Lanifera* (Fr.) Gremli. The name was recently typified [92]. It is characterized, as other members of the *H. laniferum* group, by a densely hairy (eriopodous) plant base while its other parts are nearly glabrous or glabrescent. *Hieracium spathulatum* Scheele (≡*H. laniferum* subsp. *spathulatum* (Scheele) Zahn) might be the hybrid of *H. laniferum* and *H*. *neocerinthe* (its identity is not clear and no type of material is known); both species apparently coexist in the Catalan Pre-Coastal Range, mainly S of the Ebro River, and intermediate populations are reported [73]. Only triploid counts are reported for *H*. *spathulatum* [47]. More detailed taxonomic investigation is needed to elucidate relationships in this group of species. *Hieracium laniferum* was not included in any molecular analyses.

- ***H. lawsonii*** Vill. *s*.*l*. (incl. *H*. *rupicaprinum* Arv.-Touv. & Gaut., *H*. *flocciferum* Arv.-Touv.)

This group of species is recorded from France, Italy, and Spain [68,69,73,74], showing the highest diversity in the Pyrenees [73,74]. It typically grows in rocky calcareous habitats between 400 and 2200 m a.s.l. [69,73,74]. There is considerable uncertainty regarding species delimitation and their relationship in the group of taxa included in the *H*. *lawsonii* series [74]. This species has been traditionally included in sect. *Cerinthoidea*. Morphologically two sub-groups can be separated. First, the *H*. *sericeum* Lapeyr. group (distinct from *H*. *cerinthoides*, see *H*. *venascanum* Arv.-Touv. & Gaut., *H*. *mixtiforme* Arv.-Touv.) has lanate leaves and extremely variable indumentum on the involucre and an almost identical habitus (incl. *H*. *andurense* Arv.-Touv. (≡*H*. *candidum* Scheele subsp. *andurense* (Arv.-Touv.) Mateo, Egido & Gómiz, *H*. *candidum*, *H*. *eriomallum* J.-M.Tison & Greuter, *H*. *phlomoides* Froel.); these species may represent extreme morphs of a polymorphic species possibly including diploid lineages. Secondly, the *H*. *lawsonii* group has less densely hairy to subglabrous leaves and a more uniform appearance. In the Alps, *H*. *lawsonii* is known only with triploid counts [63]. The diploid counts reported in the literature refer to *H*. *rupicaprinum* and *H*. *flocciferum* [93], collected in the Eastern Pre-Pyrenees, which are apparently related to *H*. *lawsonii s.l.* group [74]. The former species is reputed to be the intermediate *H*. *candidum*-*H*. *phlomoides* [81], or the hybrid of *H*. *hastile* Arv.-Touv. & Gaut. (≡*H*. *laniferum* subsp. *hastile* (Arv.-Touv. & Gaut.) Mateo, Egido & Gómiz) and *H*. *andurense* [73]. *Hieracium hastile* forms a distinct group of species, related to *H*. *lawsonii* series, with subglabrous, dentate, undulate, coriaceous leaves and may include diploid lineages too considering its extreme variability and the numerous taxa linked to this taxon [73]. *Hieracium flocciferum* was temporarily placed under *H*. *cryptanthum* Arv.-Touv. & Marcailhou, which is suspected of being an apomictic, in Flora Gallica [74]. Also, the single diploid count reported for *H*. *cordifolium* subsp. *neocerinthe* (Fr.) Zahn (≡*H*. *neocerinthe*) [93] is tentatively included here considering that currently no type of material is known, and the identity of this species is not clear, while it shows affinities with *H*. *lawsonii* [74]. Traditionally this species was included in *H*. *cordifolium* but most of the specimens from the Iberian Peninsula previously identified as *H*. *cordifolium* are probably referable to *H*. *neocerinthe* [76]. The type specimen of *H*. *cordifolium* is unrelated to the species to which the name was generally applied but instead belongs to *H*. *umbellatum* [94]. Therefore, the counts published for *H*. *cordifolium* [18,37,47] are included under this broadly defined taxon. In conclusion, the precise identity of the analyzed plants is not certain, and these records are provisionally referred to a widely circumscribed *H*. *lawsonii* until further evidence. Molecular analyses placed *H*. *cordifolium* in the “Western” clade among all the other Pyrenean species sharing the same cpDNA haplotypes [15,34,35].

- ***H. legrandianum*** Arv.-Touv. (incl. *H*. *amplexicaule* L. sensu auct.)

The native range of this species is limited to the Pyrenees and NE Spain (Northeastern Catalonia: Northern Catalan Mountains and Osona), where it grows in rocky places between 650 and 1400 m a.s.l. [76,81]. The only diploid record [47] should probably be referred to as this taxon, despite it being mentioned as *H. amplexicaule*, which has a much broader distribution and includes only tri- or tetraploids cytotypes [63]. Until recently [7,8,9,81,89], it was placed within *H*. *cordatum*, as *H*. *cordatum* subsp. *legrandianum* (Arv.-Touv.) Zahn, and the former species can be regarded as a morphological intermediate between *H. amplexicaule* and *H. gouanii* [95]. It can be placed in sect. *Amplexicaulia* (Griseb.) Scheele. It is included in the series of *H*. *amplexicaule*, from the Eastern Pyrenees, which comprises numerous taxa attributable to the ancestral polymorphic taxon *H*. *legrandianum* [74]. For some considerations on *H*. *cordatum* see under *H*. *gouanii*. Phylogenetically, the related *H*. *amplexicaule* is placed among the “Western” clade in the “Pyrenean” subclade together (with the other Pyrenean clade) with triploid accession of *H*. *cerinthoides* (triploid cytotype), *H*. *cordifolium*, the apomictic *H*. *candidum*, and *H*. *gymnocerinthe* all sharing the “Pyrenean” cpDNA haplotypes with some minor differences and apparently no link to any other taxa [15,34,35].

- ***H. lucidum*** Guss.

Endemic to Italy, this species is known only from four sites in NW Sicily [96,97,98] where it typically grows on NW-facing calcareous rocks and vertical cliffs, between 200 and 700 m a.s.l. [70]. All counts record *H. lucidum* as diploid [23,43,99,100,101]. Placed in sect. *Lucida* Stace & P.D.Sell, this late flowering species share some similarities with the widespread and rather variable *H. racemosum* Waldst. & Kit. ex Willd. *s*.*l*. and other closely related taxa. Traditionally treated as a distinct species, *H. cophanense* Lojac. (≡*H. lucidum* subsp. *cophanense* (Lojac.) Greuter) might correspond to a geographically isolated morph differing from typical *H. lucidum* only by the presence of more or less abundant simple hairs on stems and leaf margins, midrib, and lower face [96]. Considering the restricted area of occurrence with few locations and the declining number of individuals and quality of habitat, this species has been assessed according to the IUCN criteria [102] as Critically Endangered (CR B1ab(iii,v) [96,97]. A molecular study recently demonstrated that the two morphs are better treated as one single taxon as they share the same cpDNA haplotypes and the same distinct ETS variants (but with a pattern of unequal ratios) [35]. The species resulted in the “Western” clade and presented numerous and largely unique polymorphisms [15,34,35].

- ***H. naegelianum*** Pančić

The species occurs in the central part of the Balkan Peninsula (Serbia and Montenegro, Macedonia, Albania, Greece, Bulgaria) [68] and Central Italy where it is recorded only from the mountains of the Abruzzo region [69]. It is found in a variety of habitats usually with a thin soil layer such as stony alpine meadows and crevices, on cliffs, rocks, and gravel, on limestone, sandstone, and schist, restricted to alpine or subalpine levels [69,82]. Three diploid populations are reported from Macedonia [49,50]. The recently described *H. renatae* Szeląg [49] differs only in hairiness and size of flower heads (glabrous and smaller in *H*. *renatae* versus hairy and larger in *H*. *naegelianum*); in consideration of the morphology, genetic background, and ecology, it has been synonymized with *H*. *naegelianum* [35]. It has a unique morphology, forming underground rhizomes, that allows the plant to develop into dense clumps or tufts, which is an atypical habit for *Hieracium s*.*s*. This species is placed in its own section, recently instituted (sect. *Naegeliana* Zahn ex Szeląg) [103]. Triploid counts are also recorded, e.g., [18,43]. The Italian plants have been separated at subspecific level as *H. naegelianum* subsp. *andreae* (Degen & Zahn) Zahn [7,8,9,69] on the basis of the overall denser indumentum. Similar morphs occur in the Balkan Peninsula and the variation appears continuous, hence probably the two disjunct populations should be merged in one taxon [82]. The triploid accession included in the molecular analyses belongs to the “Eastern” clade and showed unique cpDNA haplotypes as well as some unique polymorphisms; its isolated position in the “Eastern” clade and the unusually low DNA size might be due to introgression from a “Western” species [15,34,35].

- ***H. petrovae*** Vladimir. & Szeląg

This recently described species is endemic to S Bulgaria and is currently known only from a few localities in the Central Rhodope Mountains, although it could possibly occur in the neighboring mountains of Greece [46]. This calciphilous chasmophyte grows in rock crevices, cliffs, and screes of limestone in dry sites at middle altitudes, between 1000 and 1200 m a.s.l. [46]. Only diploid counts are reported [18,46,104]. This taxon shows clear affinities with *H*. *pannosum s*.*l*. (see sect. *Pannosa* [7,8,9,10]) from which it differs by the sparser indumentum, the smaller generally dentate leaves and the narrower smaller capitula; *H*. *pannosum s*.*s*. is known only with polyploid cytotypes, e.g., [93], and has the broadest distribution (Balkans to Anatolia) among the members of the section, which includes *H*. *waldsteinii* Tausch and *H*. *gymnocephalum*. Molecular analyses place *H*. *petrovae* into the “Eastern” clade sharing some polymorphisms with *H*. *pannosum*; cpDNA haplotypes resulted identical to those of *H*. *kittanae* and *H*. *pannosum*, the latter species is suspected to have originated from *H*. *petrovae* [15,34,35].

- ***H. plumulosum*** A.Kern. (≡*H*. *waldsteinii* subsp. *plumulosum* (A.Kern.) Freyn)

This species is distributed across the Western Balkans (Albania, Montenegro, and ex-Yugoslavia) [68]. It typically grows in rock crevices and ledges on various lithologies in the montane and lower alpine zones [82]. Only two diploid counts have been recorded so far [18,48] and conservatively the record of *H*. *waldsteinii* subsp. *suborieni* Zahn [93] is placed under this taxon until further evidence. The characteristic plumose indumentum is reminiscent of *H*. *tomentosum* L. although this feature does not imply any relationships, instead, this character is reputed to be the result of adaptation to dry climate. Occasionally confused with *H*. *tomentosum* (possibly due to the synonym *H*. *lanatum* Waldst. & Kit. ex Willd.), it is readily distinguished by the mostly glandular indumentum of peduncles and involucres, and the different cauline leaves disposition, which are closely spaced and limited to the lower half of the stem. *Hieracium thapsiforme* R.Uechtr. ex Nägeli & Peter (≡*H*. *waldsteinii* subsp. *thapsiforme* (R.Uechtr. ex Nägeli & Peter) Freyn) from Montenegro, traditionally treated as a distinct taxon, separated by the density of the stem indumentum, is considered conspecific with *H*. *plumulosum* (see sect. *Pannosa*) as a certain degree of morphological variability is typical for sexual species [105]. The diploid accession included in molecular analyses resulted in an interclade hybrid, with predominance of the “Eastern” clade contribution, which did not fit in any particular species subgroup and displayed a highly reticulate history; this taxon presented a unique cpDNA haplotype divergent from all other species and partly congruent with *H*. *gymnocephalum* [15,34,35].

- ***H. pojoritense*** Woł.

This species is endemic to NE Romania, found only in the Eastern Carpathians and it is known from ca. 15 localities situated in three mountain massifs and their surroundings [106,107]. It is a calciphilous species confined to limestone or conglomerate rocks and screes usually in moist sites, between 630 and 1300 m a.s.l. [106,107]. Only diploid counts are reported [23,28,107]. This taxon, placed in sect. *Italica* (Fr.) Arv.-Touv., which includes late flowering species, resembles *H*. *racemosum s.l.* but it is clearly distinguished by its morphology, more developed rhizome and well-defined basal rosette with few small cauline leaves, and ecology, growing in more rocky and exposed habitats (instead of woodlands and clearings) and having a much shorter flowering season (August to September vs. July to November). It is interpreted as an intermediate between *H*. *sparsum* Friv. and *H*. *racemosum* [106] or according to Zahn *H*. *sparsum* and *H*. *sabaudum* L. [7,8,9]. The accession included in molecular analyses presented a high number of unique polymorphisms, partly shared with the “*H*. *umbellatum”* clade (including *H*. *racemosum*) together with some *H*. *alpinum*-specific synapomorphies; cpDNA corresponded to the latter species (as well as ETS ribotype). Considering that this is presumably an old species occurring in a renowned glacial refugium, the lack of any apparent resemblance with *H*. *alpinum* might suggest the influence of a closely related extinct ancestral taxon. In this regard, the cpDNA haplotype characteristic of *H*. *alpinum* was found in *H*. *sparsum* supporting its contribution as one of the supposed parental species [15,34,35].

- ***H. porrifolium*** L.

This species is mainly distributed in the Southern and Eastern Alps including Austria, Slovenia, and Italy, where it is recorded also from the Central Apennines in the Apuan Alps [68,69]. It is a rupicolous and calciphilous species typically growing in crevices on limestone slopes and rocky or arid pastures in exposed sites, between 100 and 500 m a.s.l. [68,69]. Only diploid counts are reported [23,78,108,109]. It has been recently lectotypified on Boccone’s illustration, and an epitype was designated from the Austrian Alps [110] (pp. 890–891). This is a quite distinct and morphologically uniform species distinguished by the narrowly linear long leaves with a glaucous tinge and entire margins, involucres usually only with stellate hairs, and few sparse glandular or simple hairs. This species is likely involved in the origin of many members of sect. *Drepanoidea* (possibly resulting from the crossing with *H*. *dollineri*, e.g., *H*. *bupleuroides* C.C.Gmel., *H*. *glaucum*). Merxmüller [43] (p. 193) records a spontaneous interspecific cross between two diploid taxa, *H*. *porrifolium* and *H*. *umbellatum*, cultivated in Munich botanical garden which resulted in the diploid hybrid *H*. *leiocephalum* Bartl. ex Griseb.; no counts were made for this species, and more data are needed to confirm this putatively additional diploid taxon (or possibly an occasional hybrid), which is not included in the present list. Molecular analysis placed this taxon in the “Eastern clade”, forming the “*H*. *porrifolium”* subclade, and identified a particular cpDNA haplotype [15,34,35].

- ***H. prenanthoides*** Vill. *s.s*.

The diploid cytotype is currently known only from France in the SW Alps [74] but it is very likely to occur also on the Italian side although no counts are reported at present. It grows in woodlands, their margins and clearings of the mountain region typically on calcareous soils, between 800 and 2000 m a.s.l. [74,111]. Diploid counts come from a restricted geographic range [23,37,112,113], coinciding with a glacial refuge area. These relict populations appear morphologically fairly uniform, usually with more than 10 strongly auriculate cauline leaves, small densely glandular capitula, and livid stigmas [74]. *Hieracium prenanthoides s*.*l*. counts numerous polyploids taxa (see in [63]) distributed across the European mountains, the Caucasus, and neighboring areas (reaching Central Asia and Siberia); these species have been interpreted as intermediates with numerous other species (e.g., *H*. *umbellatum*) and are placed in sect. *Prenanthoidea* W.D.J.Koch. Molecular analyses confirm the complex history of reticulation, involving different clades and repeated hybridization events, underlying polyploids; the diploid accession resulted in an interclade hybrid, with a “Western” clade origin, having “lost” most of the “Eastern” clade sequences [15,34,35].

- ***H. racemosum*** Waldst & Kit. ex Willd. *s*.*l*.

This is a widespread species present in most of continental Europe (excl. Spain) reaching Ukraine and Turkey in the east [68]. It is typically found in various types of woodland and their margins and clearings, growing preferably on slightly acidic or neutral substrates, between 100 and 2000 m a.s.l. [68,69]. Two diploid counts are reported [61,79], otherwise this species is known only with triploid cytotypes [63]. One of the analyzed populations [79] presented mixed cytotypes and has been ascribed to *H*. *racemosum* subsp. *leiopsis* Murr & Zahn. Traditionally placed in sect. *Italica*, it must be considered that this is an extremely polymorphic and variable species. Molecular analysis retrieved it as an interclade hybrid with cpDNA haplotypes shared with the “*H*. *umbellatum”* group; it is suspected of polytopic origins, involving different additional taxa, which might explain its high variability [15,34,35].

- ***H. recoderi*** de Retz

The native range of this species is NE Spain [68,73], it is known only from the Eastern Pre-Pyrenees where it grows in rocky places between 900 and 1500 m a.s.l. Only two counts from the locus classicus are reported [18,37]. This apparently distinctive species has been overlooked in the past and was only described in the late 1970s [113]. It is regarded as the probable ancestor for several taxa endemic to the Northeastern Iberian Peninsula [73]. *Hieracium queraltense* de Retz is thought to be the hybrid of *H*. *neocerinthe* and *H*. *recoderi* [73,113]. This taxon is here provisionally considered as a distinct species, but more detailed investigations are needed as this species might actually be conspecific with *H*. *gouanii* (sect. *Cerinthoidea*) and included in its range of variability. According to molecular analyses, this species belongs to the “Pyrenean” clade, it shares the same cpDNA haplotypes with *H*. *ramondii* [15,34,35].

- ***H. sparsum*** Friv. *s.s*.

This diploid species is distributed across Southeastern Europe and Northern Anatolia, with scattered relict stations in the E Alps, E Sudetes, W and E Carpathians, and in W Anatolia [103]. It is found in open coniferous woodlands, grassy slopes, subalpine and alpine meadows and it appears confined to acidic bedrock or lime-poor soils, usually from the upper montane zone above 1500 m a.s.l. [82,103]. Only diploid counts are known for *H*. *sparsum s*.*s*. [23,37,48,50,51,83,104,114,115] ([114] on plants of unknown origin). This species is placed in sect. *Cernua* R.Uechtr. The *H*. *sparsum* group (or *H*. *sparsum s*.*l*.) [7,8,9] is a morphologically rather uniform complex of polyploid species, e.g., [79,115], with two main diversity centers located in the Balkans and in the Caucasus. Molecular analyses placed the diploid accessions at the base of the “Eastern” clade, suggesting it might be a quite old taxon, and retrieved some unique polymorphisms; cpDNA is similar and possibly derived from an “*H*. *alpinum*” or its ancestor (see notes on *H*. *pojoritense*) [15,34,35].

- ***H. stelligerum*** Froel.

This species is endemic to Southern France in the Languedoc region; it typically grows on rocks or in rocky habitats, up to 700 m a.s.l. [74]. The few recorded counts are diploid [18,23]. Placed in sect. *Stelligera* Zahn, it is readily distinguished by the presence of stellate hairs on the upper side of leaves [74]. *Hieracium stelligerum* series includes a single diploid and three scarcely distinguishable apomictics [74]. It is considered a relict species being known only from a few populations in a circumscribed area (triangle Montpellier-Montélimar-Castellane). Molecular analyses place this taxon in the “Western” group with the closely related species *H*. *bifidum* (or “bifidoid” species), sharing the same cpDNA haplotypes of the latter [15,34,35].

- ***H. tomentosum*** L. *s.s*.

Endemic to the Western Alps, this species is found in Italy, France, and Switzerland; it typically grows on calcareous rocks, between 500 and 2300 m a.s.l. [69,74,111]. A single diploid count is present in the literature, where also a single triploid count is reported [18]. The diploid populations appear restricted to the Upper Roya Valley both on the French and Italian sides, possibly extending to other mountains of the inner Ligurian Alps (pers. observation). It has been recently lectotypified on Dillenius’s illustration and an epitype was designated from the Col de Tende/Colle di Tenda area at the France–Italy border where the counted diploid populations occur [110] (pp. 892–893). It is placed in sect. *Andryaloidea* Monnier. The *H*. *tomentosum* series [74,111] is distinguished by the strongly plumose hairs forming a dense felty whitish indumentum all over the plant and includes a few species, which are currently under study, distributed from the W Alps to the Central Apennines reaching Abruzzo region [69]. *Hieracium andryaloides* Vill. (≡*H*. *tomentosum* subsp. *andryaloides* (Vill.) Nägeli & Peter) shows an almost identical indumentum, although often shorter and more compact, and expresses a surprising degree of morphological variation in the Pre-Alps of Southern France suggesting the possible existence of diploid lineages [111]; its relationship with *H*. *tomentosum s*.*s*. should be studied more in-depth. Molecular analyses place this species in the “Western” clade and most of its polymorphisms are unique and substitution characteristics suggest possible relations with some of the Pyrenean species [15,34,35].

- ***H. transylvanicum*** Heuff.

This is a widespread species, from Central and Eastern Europe to Ukraine, recorded from Albania, Austria, Bulgaria, Czechoslovakia, Poland, Romania, Ukraine, and ex-Yugoslavia [68,106]. It typically grows in spruce and beech mountain forests [60]. Only diploid cytotypes are found across its range [12,18,23,26,28,37,44,48,50,53,60,71,104,116] ([26] garden plant of unknown origin, sub *H*. *rotundatum* Kit. ex Schult.). It is traditionally placed in sect. *Vulgata* (Griseb.) Willk. & Lange. Morphologically, *H*. *transylvanicum* is relatively uniform but somewhat variable in the growth form especially considering the size of the plant and the presence or absence of cauline leaves. Molecular analyses placed accessions into the “Western” clade despite its eastern distribution [15,34,35]. This might be the effect of ancient introgression from a member of the “Western” clade resulting in complete homogenization, or on the contrary the species may have originated in W Europe, where it later went extinct, and spread towards the east surviving just here the glaciations and apparently leaving no evident traces (or possibly in the *H*. *lachenalii* Suter group); the latter scenario is supported by its unique cpDNA haplotypes and the unusually high DNA size although the former hypothesis seems more plausible given its current distribution [15,34,35].

- ***H. umbellatum*** L.

This species has the largest distribution of all *Hieracium s.s.* species encompassing the temperate Northern Hemisphere, from Eurasia to North America and Greenland [68]. It has an ample ecological tolerance, growing from coastal areas, in the northern countries, to the upper montane or sub-alpine meadows, between 0 and 2000 m a.s.l. [68,69,73,74]. It is known mostly with numerous diploid counts [17,18,23,24,28,37,44,79,104,117,118,119,120,121,122,123,124,125,126,127,128,129,130,131,132,133,134,135], although some counts resulted in triploid (see respective references in [63]). The records include specimens ascribable to this hypervariable species but reported with dubious identification: sub *H*. *conicum* Arv.-Touv. [44], sub *H*. *hryniaviense* Woł. [28], sub *H*. *laevigatum* Willd. [132], sub *H*. *sabaudum* [129], sub *H*. *laurinum* Arv.-Touv. (=*H*. *vasconicum* subsp. *laureolum* (Arv.-Touv.) Greuter) [134]. Until further evidence is provided, these records are tentatively included in this peculiar species. *Hieracium umbellatum* was recently typified on original material [136]. It is usually a tall-growing species (shorter and more compact at higher elevations), with numerous cauline leaves variable in shape and a scarce indumentum of mostly stellate hairs throughout the plant. It is reportedly extremely polymorphic, so some of its putative subspecies develop differently shaped leaves (narrow or broad) in subsequent years of cultivation [128,137,138] ([137] p. 624). Thus considered, sect. *Hieracioides* Dumort., in which this taxon is placed, should be carefully investigated and consequently revised to reflect the natural range of variation of sexual species. The diploid accessions included in molecular analyses were placed in the “Eastern” clade forming the “*H*. *umbellatum”* clade together with *H*. *canadense* Michx., likely conspecific from North America [139] (USA), and *H*. *eriophorum*; all shared the same characteristic cpDNA haplotypes [15,34,35].

- ***H. valdepilosum*** Vill. subsp. ***subsinuatum*** (Nägeli & Peter) Zahn

This species is present in the Alps in Central Europe, Austria, France, Germany, Italy and Switzerland [68]. It usually grows in the subalpine belt in meadows and rocky habitats on calcareous substrates [69,74]. The first and only diploid count reported so far comes from Austria [78]. Placed in sect. *Villosa* (Griseb.) Gremli, *H*. *valdepilosum s*.*l*. has been traditionally interpreted as the intermediate between *H*. *villosum* Jacq. and *H*. *prenanthoides*, while subsp. *subsinuatum* is placed in subgrex *H*. *oligophyllum* Zahn and is considered morphologically closer to *H*. *villosum* [7,8,9]; this group of species has been recently assimilated to *H*. *morisianum* Rchb.f. (=*H*. *pilosum* Schleich. ex Froel. subsp. *villosiceps* Nägeli & Peter ex Gottschl.), although there is apparently no clear demarcation with *H valdepilosum* [74,111]. The finding of diploid cytotypes in this group of species indicates the need for more accurate investigation. *Hieracium valdepilosum* series includes some extremely variable and scarcely distinct taxa characterized by the hypo- or aphyllopodous habit and the numerous cauline leaves with more or less numerous hairs on their upper side [74,111]. It has not been included in any molecular studies yet.

- ***H. virgaurea*** Coss. (≡*H*. *racemosum* subsp. *virgaurea* (Coss.) Zahn)

This diploid species is relatively widespread, mainly in peninsular Italy but lacking in Sicily and Sardinia; it is a woodland species growing in fresh or moist sites with neutral to acidic substrates, often associated with chestnut groves, various woodlands and clearings [68,69]. It is present in Corsica, while the plant has been erroneously recorded from mainland France, Maritime Alps, or has possibly gone extinct there [74]. Surprisingly, very few counts are reported [43,61] ([43] on plant of unknown origin). Placed in sect. *Italica*, this late flowering species was traditionally treated as a subspecies of *H*. *racemosum s*.*l*. [7,8,9,69], from which it is distinguished mainly by the clearly petiolate vernal leaves, the scarce or extremely reduced involucres’ indumentum, and the pale-tinged achenes (reddish or brownish in *H*. *racemosum s*.*l*. when ripe). At present, it has not been included in any molecular studies.

- ***H. vranceae*** Mráz

This recently described species is endemic to Romania and is currently known only from three close sites in the Vrancea Mountains in the Curvature Sub-Carpathians [35]. It is a calciphilous species reported growing on limestone, conglomerate rocks, and screes around 600–700 m a.s.l. [35]. It is a rather distinctive small plant with conspicuously glaucous, subcoriaceous leaves and deeply branched sinflorescence with branches bearing only one capitulum (monocephalous). It has not been assigned to any specific section, though its morphology suggests its placement among the late flowering species of sect. *Italica*. This species appears strictly associated with relictual calcareous rocky habitats, similarly to *H*. *pojoritense* but apparently preferring drier sites. Molecular analyses place this taxon in the “Eastern” clade and support its distinctiveness from all other species. Considering the small population size (estimated to have less than 1000 mature individuals) this taxon has been assessed as vulnerable (VU) according to criterion D1 of IUCN. Phylogenetic analyses suggest this species might represent a new lineage in the genus [35].

## 6. *Hieracium* Hotspots

**Figure 1:**
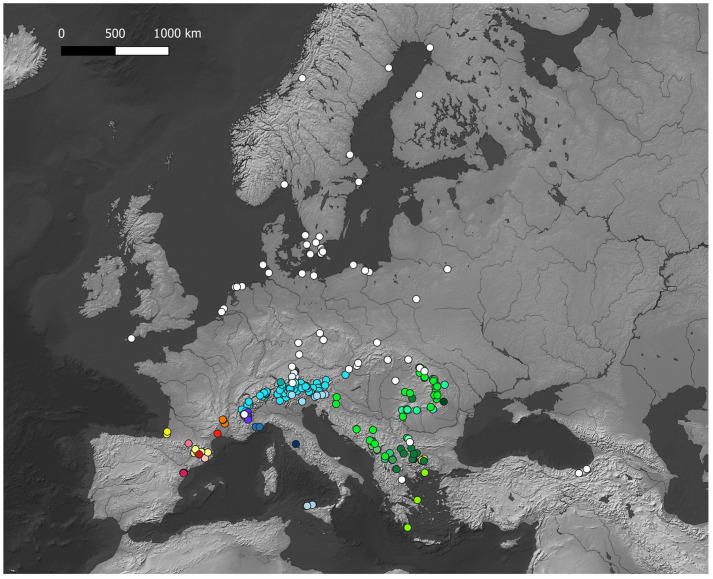
Map showing the distribution of the diploid chromosome counts of *Hieracium* reported in this review (for the legends see Figure 2, Figure 3, Figure 4 and Figure 5).

**Figure 2:**
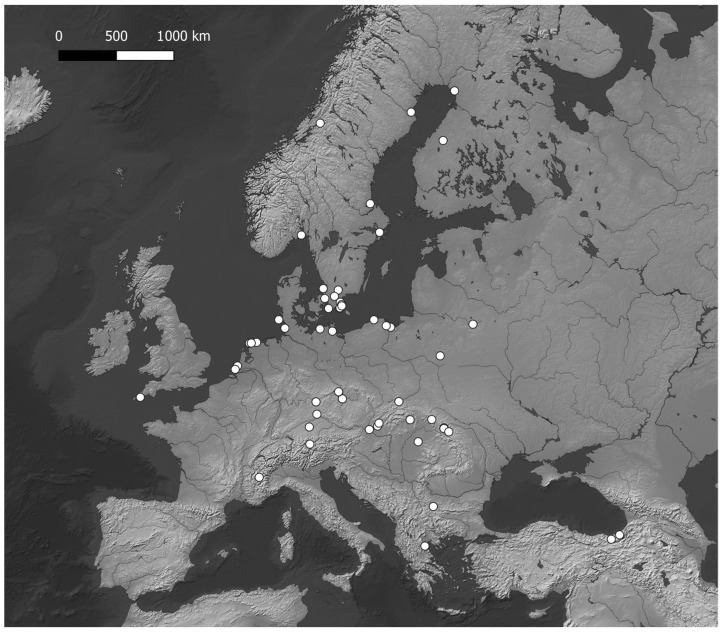
Map showing the distribution of the diploid chromosome counts of *H*. *umbellatum*.

**Figure 3:**
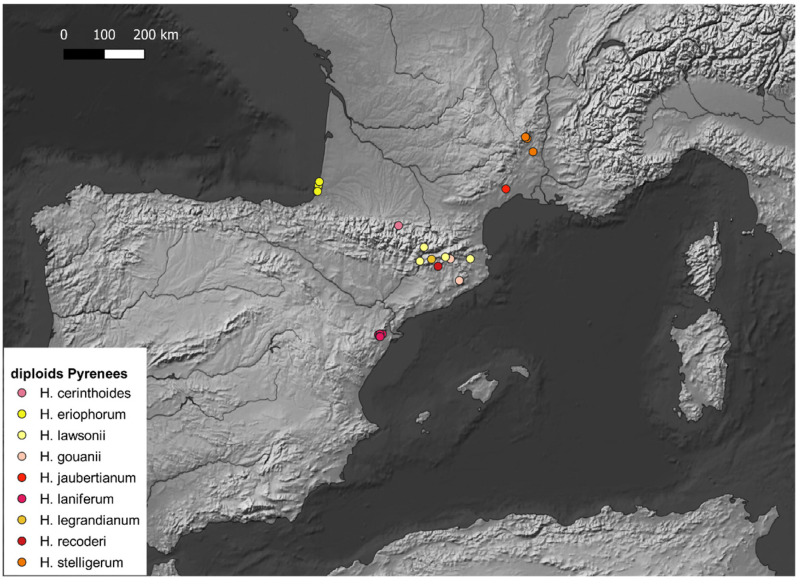
Map showing the distribution of the diploid chromosome counts from SW France and Spain.

**Figure 4:**
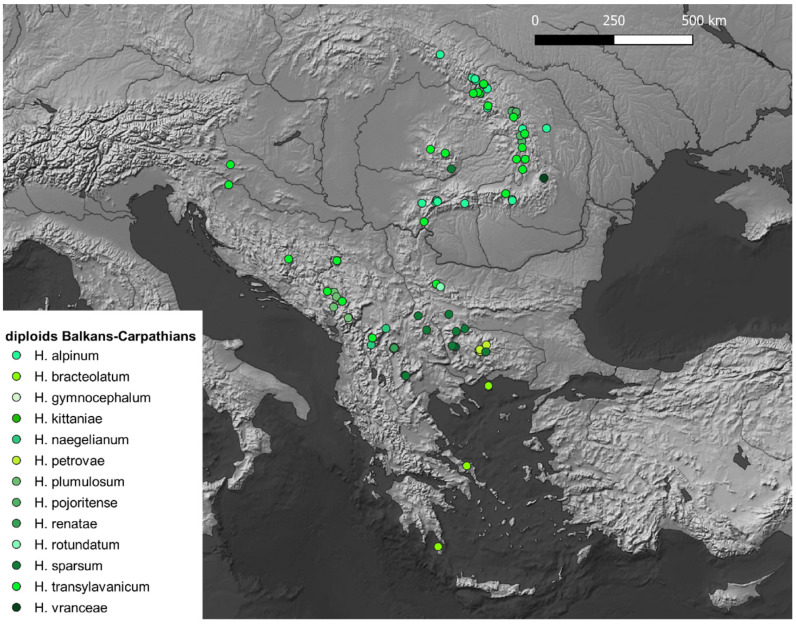
Map showing the distribution of the diploid chromosome counts from the Balkan Peninsula and Eastern Europe (incl. Ukraine).

**Figure 5:**
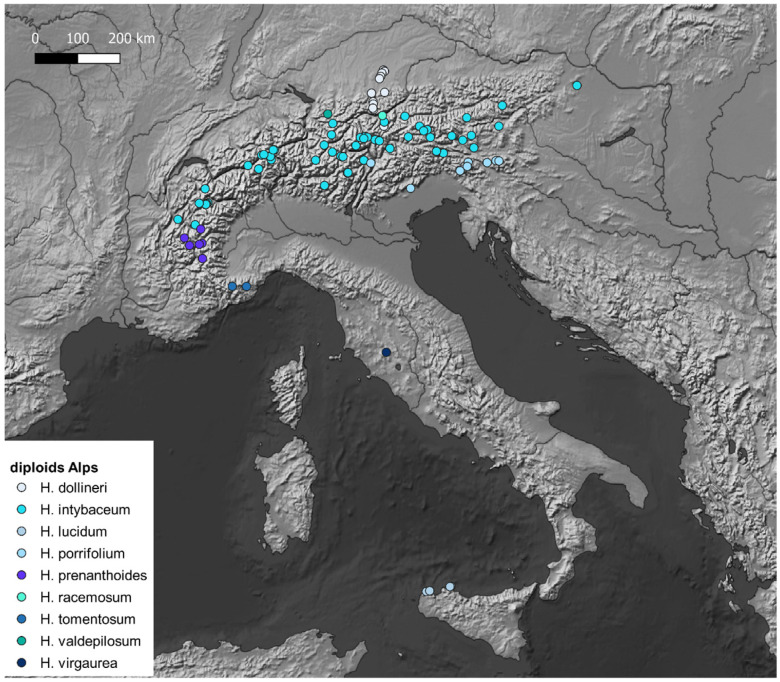
Map showing the distribution of the diploid chromosome counts from the Alps and Italy.

Records of diploid *Hieracium* have been georeferenced (see Supplementary Material S1) and projected on maps with QGis to highlight the hotspots of diploid *Hieracium s.s*. diversity. The maps (Figure 1, Figure 2, Figure 3, Figure 4 and Figure 5) show the distribution of the currently known diploids counts. As supposed, the majority of the taxa appear confined to the principal mountain ranges in Europe and neighboring countries (Alps, Pyrenees, Carpathians, etc.), with the exception of *H*. *bracteolatum* (Figure 4) and *H*. *lucidum* (Figure 5), which are known only from insular environment (Aegean Islands and Sicily, respectively). *Hieracium jaubertianum*, *H*. *stelligerum* (Figure 3), and *H*. *virgaurea* (Figure 5), on the contrary, seem to be restricted to the Mediterranean area with different ecological requirements and climatic ranges. 

**Figure 6:**
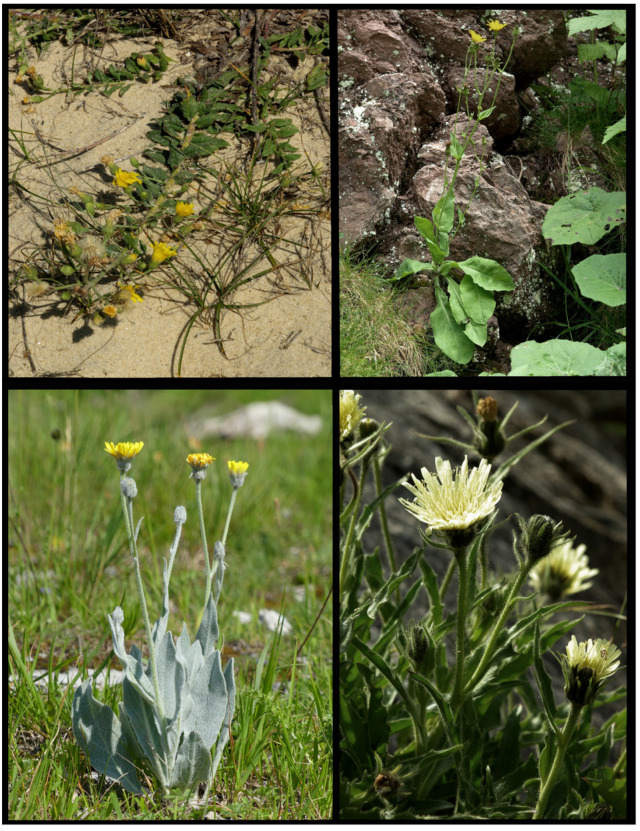
Picture of some diploid *Hieracium* species, clockwise form the upper left: *H*. *eriophorum*, *H*. *gouanii*, *H*. *intybaceum* (photographs of J.-M. Tison), *H*. *tomentosum* (photograph of S. Orsenigo).

*Hieracium cerinthoides*, *H*. *elisaeanum*, *H*. *gouanii* (Figure 6), *H*. *laniferum*, *H*. *lawsonii s*.*l*. (see *H*. *lawsonii* series), *H*. *legrandianum,* and *H*. *recoderi* are known only from the Pyrenees and environs. *Hieracium eriophorum* (Figure 6) appears unique in being confined to the coastal environment in the Southern Atlantic Coast of France (Figure 3).

On the other hand, *H*. *alpinum*, *H*. *gymnocephalum*, *H*. *kittanae*, *H*. *naegelianum*, *H*. *petrovae*, *H*. *plumulosum*, *H*. *pojoritense*, *H*. *sparsum*, *H*. *transylvanicum,* and *H*. *vranceae* are all species limited to the Balkan region or with diploid cytotypes known only from this area (Balkan Mountains, Carpathians, Rhodopes, etc.) (Figure 4).

*Hieracium dollineri*, *H*. *intybaceum* (Figure 6), *H*. *jaubertianum*, *H*. *porrifolium*, *H*. *prenanthoides*, *H*. *racemosum s*.*l*., *H*. *stelligerum*, *H*. *tomentosum* (Figure 6), and *H*. *valdepilosum s*.*l*. are all species with their distribution centered on the chain of the Alps and Prealps (Figure 5).

Overall, the highest diversity of diploid *Hieracium s.s.* coincides with the European glacial refuges and these areas should be meticulously surveyed in order to ameliorate our knowledge of diploids.

## 7. Final Considerations/Remarks

This review aims to present a baseline for further studies on diploids given the lack of an updated and comprehensive database of cytological data for subgen. *Hieracium*.

The list of diploids here presented includes 31 species; this number is provisional considering that for some taxa, data are scarce and/or outdated (e.g., *H*. *bifidum*, *H*. *cerinthoides*, etc.). Additionally, Pyrenean and Iberian taxa are probably taxonomically over-split and more detailed taxonomic investigations are needed [35,74,76]. Similarly, the whole *H*. *waldsteinii-plumulosum*-*gymnocephalum* group needs a revision [84,85]. Solely for *H*. *alpinum*, *H*. *intybaceum*, *H*. *transylvanicum*, and *H*. *umbellatum* numerous counts from large parts of their ranges have been published; on the contrary, for *H*. *jaubertianum*, *H*. *tomentosum*, and *H*. *virgaurea* a single count each has been published so far, and these taxa will need more accurate investigations to understand their effective distribution and potential cytotype patterns.

The diploid count for *H*. *elisaeanum* Arv.-Touv. ex Willk., made by Merxmüller on plants of unknown origin and reported in a posterior publication [140], was excluded; the identity of the analyzed plant is unclear, and only triploid counts have been reported for this taxon so far [47]. Additionally, the count relative to *H*. *pseudocorymbosum* Gremli (sub *H*. *pseudocorymbosum* subsp. *petryanum* (Zahn) Zahn) [79] is not included in the list as the identity of the analyzed taxon is dubious; the same applies to *H*. *virgicaule* Nägeli & Peter [141] and *H*. *speciosum* Hornem. [27]. Further investigation will confirm the placement of these species and their ploidy level.

Other diploid species are known from Asia and the Eastern part of Russia: *H*. *filifolium* Üksip [142], *H. korshinskyi* Zahn [143], *H*. *narymense* Schischk. & Serg. [144], and *H*. *virosum* Pall. [145]. The aforementioned species were not included in the present review, considering they do not or only marginally occur in Europe, data are scarce, and publications are hardly accessible. More detailed analyses are needed before some considerations can be made. It is likely that these species, together with the ones reported in this paper, contributed at least partly to the European *Hieracium* species diversity, especially in its Central and Eastern range.

Furthermore, additional species occurring in Europe are suspected of being diploid and are currently under study. Such species include *H*. *caesioides* Arv.-Touv. *s*.*l*., *H*. *nemorense* Jord. (≡*H*. *murorum* subsp. *nemorense* (Jord.) Zahn), *H*. *tenuiflorum* Arv.-Touv *s*.*l*. 

As evidenced by this work, large gaps in the knowledge of *Hieracium s*.*s*. are still present, and more detailed studies are needed to investigate the complex dynamics that have originated due to the outstanding diversity that we observe nowadays. Considering the vast number of species and the astonishing degree of variability, it is expected that more diploids will be discovered when a more accurate and systematic screening of various taxa is carried out.

## Data Availability

Data sharing is not applicable.

## Supplementary Materials

The following supporting information can be downloaded at: https://www.mdpi.com/article/10.3390/plants14071057/s1, Table S1: dataset diploid Hieracium.
