# Supplementary material for: Preliminary Review of the Diploid Taxa in Hieracium s.s
Source: Plants (Basel). 2025 Mar 29;14(7):1057. doi: 10.3390/plants14071057 (PMC11991471; doi:10.3390/plants14071057)
Supplement: Supplementary file 1 [file plants-14-01057-s001.zip › Supplementary File S1 (diploid Hieracium dataset) final.pdf]

Species	Country	Locality	Latitude	Longitude	Reference	Notes	
Hieracium alpinum L.	Ukraine	Mt. Dragobrat	48,23237	24,23176	[52]	2n=2x=18 var. *alpinum*	
Hieracium alpinum L.	Ukraine	Mt. Stih	48,25142	24,22351	[52]	2n=2x=18 var. *alpinum*	
Hieracium alpinum L.	Ukraine	Mt. Hoverla	48,16101	24,50012	[52]	2n=2x=18 var. *alpinum*	
Hieracium alpinum L.	Ukraine	Khrebet Breskul mountain range	48,14944	24,50258	[52]	2n=2x=18 var. *alpinum*	
Hieracium alpinum L.	Ukraine	Mt. Pozhizhevska	48,14573	24,52157	[52]	2n=2x=18 var. *alpinum*	
Hieracium alpinum L.	Ukraine	Mt. Turkul	48,12429	24,53013	[52]	2n=2x=18 var. *alpinum*	
Hieracium alpinum L.	Ukraine	Mt. Dragobrat	48,23234	24,23176	[52]	2n=2x=18 var. *subglabrum*	
Hieracium alpinum L.	Ukraine	Mt. Dragobrat	48,23234	24,23176	[52]	2n=2x=18 subsp. *augusti-bayeri*	
Hieracium alpinum L.	Ukraine	Mt. Ungaryas'ka	48,28457	24,11235	[68]	2n=2x=18	
Hieracium alpinum L.	Ukraine	Mt. Tatul	48,27525	24,19983	[68]	2n=2x=18	
Hieracium alpinum L.	Ukraine	Mt. Ungaryas'ka	48,30002	24,10001	[26]	2n=2x=18	
Hieracium alpinum L.	Ukraine	Mt. Stih and Mt. Blyznytsya	48,23333	24,23333	[26]	2n=2x=18	
Hieracium alpinum L.	Ukraine	Mt. Tatul	48,27400	24,20000	[26]	2n=2x=18	
Hieracium alpinum L.	Romania	Mr. Barlea-Mt. Semele Mari	45,33333	22,36667	[26]	2n=2x=18	
Hieracium alpinum L.	Romania	Retezat Mts.	45,35655	22,84302	[69]	2n=2x=18	
Hieracium alpinum L.	Romania	Șaua Ciurila	45,40451	22,86557	[69]	2n=2x=18	
Hieracium alpinum L.	Romania	Valea Pietrele	45,40464	22,88563	[69]	2n=2x=18	
Hieracium alpinum L.	Romania	Rodnei Mts., Pietrosul	47,60087	24,63333	[69]	2n=2x=18	
Hieracium alpinum L.	Romania	Bistrița Mts., La Toacă	47,11030	25,84468	[69]	2n=2x=18	
Hieracium alpinum L.	Romania	Bistrița Mountains	47,12359	26,67467	[69]	2n=2x=18	
Hieracium alpinum L.	Ukraine	Mt. Hoverla-Mt. Breskul	48,15287	24,50407	[10]	2n=2x=18	
Hieracium alpinum L.	Ukraine	Mt. Pozhyzhevśka	48,14682	24,52144	[10]	2n=2x=18	
Hieracium alpinum L.	Romania	Mt. Retezat	45,37176	22,90432	[48]	2n=2x=18	
Hieracium alpinum L.	Romania	Mt. Coștila	45,42854	25,48494	[48]	2n=2x=18	
Hieracium alpinum L.	Ukraine	Mt. Pip Ivan Chornohirs´kyi	48,04583	24,62916	[35]	2n=2x=18	
Hieracium alpinum L.	Romania	Munții Bucegi	45,39821	25,49384	[28]	2n=2x=18 flow cytometry analysis	
Hieracium alpinum L.	Romania	Nedea? (Vârful Nedeia, Munții Că	45,32730	23,84665	[28]	2n=2x=18 flow cytometry analysis	
Hieracium alpinum L.	Ukraine	Mt. Pikuï (Skhidni Beskidi Mts)	48,83111	22,99588	[28]	2n=2x=18	
Hieracium bracteolatum Sm.	Greece	Taso (Θάσος), (Mt. Toumpa (Toύ	40,71995	24,66662	[49]	2n=2x=18	
Hieracium bracteolatum Sm.	Greece	Mt. Xeroboúni (Oros Xerokampia	36,37322	22,92668	[49]	2n=2x=18	
Hieracium bracteolatum Sm.	Greece	Mt. Xeroboúni (Oros Xerokampia	38,59055	23,91648	[56]	2n=2x=18	
Hieracium cerinthoides L.	France	Col de l'Escalette (Pic de l'Escale	42,92547	0,75168	[73]	2n=2x=18	
Hieracium dollineri Sch.Bip. ex Neilr.	Germania	Isarbrücke	47,68281	11,57153	[76]	2n=2x=18 subsp. *dollineri*	
Hieracium dollineri Sch.Bip. ex Neilr.	Germania	Menterschwaige	47,08054	11,55186	[76]	2n=2x=18 subsp. *dollineri*	
Hieracium dollineri Sch.Bip. ex Neilr.	Germania	Großhesseloher (Brücke?)	48,07587	11,54080	[76]	2n=2x=18 subsp. *dollineri*	
Hieracium dollineri Sch.Bip. ex Neilr.	Germania	Großhesselohe-Deisenhofen (bah	48,05358	11,56709	[76]	2n=2x=18 subsp. *dollineri*	
Hieracium dollineri Sch.Bip. ex Neilr.	Germania	Römerschanze	48,02309	11,50100	[76]	2n=2x=18 subsp. *dollineri*	
Hieracium dollineri Sch.Bip. ex Neilr.	Germania	Kloster Schäftlarn	47,96470	11,46889	[76]	2n=2x=18 subsp. *dollineri*	
Hieracium dollineri Sch.Bip. ex Neilr.	Germania	Wallgau	47,51652	11,26967	[76]	2n=2x=18 subsp. *dollineri*	
Hieracium dollineri Sch.Bip. ex Neilr.	Germania	Mittenwald-Krün (Isar)	47,47622	11,27660	[76]	2n=2x=18 subsp. *dollineri*	
Hieracium dollineri Sch.Bip. ex Neilr.	Germania	Isarschotter (ISAR-Schotter?)	47,38049	11,28272	[76]	2n=2x=18 subsp. *dollineri*	
Hieracium dollineri Sch.Bip. ex Neilr.	Germania	Scharnitz 2 km N (Brünnsteinhütt	47,40384	11,26269	[76]	2n=2x=18 subsp. *dollineri*	
Hieracium dollineri Sch.Bip. ex Neilr.	Germania	Wolfratshausen	47,92464	11,43724	[76]	2n=2x=18 subsp. *dollineri*	
Hieracium dollineri Sch.Bip. ex Neilr.	Germania	Loisachtal Bahnhof (Loisach?)	47,66817	11,22784	[76]	2n=2x=18 subsp. *dollineri*	
Hieracium elisaeanum Arv.-Touv. ex Willk.	Spain	-	-	-	[138]	2n=2x=18 no info (Merxmüller ined. In: D.M. Moore 1982)	
Hieracium eriophorum St.-Amans	France	Plage Labenne Océan	43,60184	-1,47254	[16]	2n=2x=18	
Hieracium eriophorum St.-Amans	France	Plage des Estagnots	43,68818	-1,44046	[16]	2n=2x=18	
Hieracium eriophorum St.-Amans	France	(Plage Des Sablères) Plage de Vi	43,79457	-1,41252	[16]	2n=2x=18	
Hieracium eriophorum St.-Amans	France	Seignosse (Plage des Casernes?)	43,72517	-1,43230	[41]	2n=2x=18	
Hieracium eriophorum St.-Amans	France	Plage Labenne Océan	43,60472	-1,45555	[35]	2n=2x=18	
Hieracium eriophorum St.-Amans	France	(Plage Des Sablères) Plage de Vi	43,79638	-1,40750	[35]	2n=2x=18	
Hieracium gouanii Arv.-Touv	Spain	Ripoll-Ribes de Fresser (Rio bove	42,25478	2,16361	[16]	2n=2x=18 Syn. *H* . *cordifolium* subsp. *gouanii*	
Hieracium gouanii Arv.-Touv	Spain	Ripoll-Ribes de Fresser	42,25750	2,16722	[35]	2n=2x=18 Syn. *H* . *cordifolium* subsp. *gouanii*	
Hieracium gouanii Arv.-Touv	Spain	Santa Fe del Montseny-Viladrau (	41,81956	2,40901	[41]	2n=2x=18 sub. *H* . *hispanicum* Arv.-Touv. (Syn. H. cordatum subsp. hispanicum) considered in H. gouanii! (occasional hybrid gouanii x legrandianum)	
Hieracium gymnocephalum Griseb. ex Pan	Monteneg	Mt. Maja Rosave	42,47927	19,83595	[81]	2n=2x=18	
Hieracium gymnocephalum Griseb. ex Pan	Albania	Mt. Jezercës (21 km NW of Bajra	42,42944	19,83972	[35]	2n=2x=18	
Hieracium grofae Woł.	Ukraine	Berlebashka (Берлебашка)	47,96061	24,30348	[37]	2n=2x=18 included in *H* . *alpinum*	
Hieracium intybaceum All.	Italy	Passo del Tonale, Laghetto Alvec	46,23684	10,59624	[16]	2n=2x=18	
Hieracium intybaceum All.	Austria	Arlbergpass	47,13048	10,21034	[21]	2n=2x=18	
Hieracium intybaceum All.	Switzerlan	Oberalppass (PP Oberalp)	46,65441	8,67704	[21]	2n=2x=18	
Hieracium intybaceum All.	Switzerlan	Furkapass	46,57324	8,39306	[21]	2n=2x=18	
Hieracium intybaceum All.	France	Cormet de Roselend (Gouille du (	45,68839	6,69619	[21]	2n=2x=18	
Hieracium intybaceum All.	France	Col du Petit Saint-Bernard	45,65815	6,86396	[21]	2n=2x=18	
Hieracium intybaceum All.	Italy	Mt. Monticello (Passo del Tonale	46,24500	10,60916	[35]	2n=2x=18	
Hieracium intybaceum All.	Italy	Alpi Oróbie	46,01033	9,99000	[35]	2n=2x=18	
Hieracium intybaceum All.	Austria	Mt. Gaisberg (Ötztaler Alpen)	46,85833	11,02500	[35]	2n=2x=18	
Hieracium intybaceum All.	Austria	Mt. Leßhöhe	47,23391	13,73325	[85]	2n=2x=18	
Hieracium intybaceum All.	Austria	Sankt Margarethen im Burgenlan	47,80502	16,63267	[86]	2n=2x=18	
Hieracium intybaceum All.	Austria	Schoberpass	47,45009	14,66671	[86]	2n=2x=18	
Hieracium intybaceum All.	Austria	Millstatter alpen (Obermillstätter	46,83217	13,62694	[86]	2n=2x=18	
Hieracium intybaceum All.	Austria	Obergurgl	46,89299	11,03568	[86]	2n=2x=18	
Hieracium intybaceum All.	Switzerlan	Col du Stelvio (Passo dello Stelvi	46,52947	10,45303	[86]	2n=2x=18	
Hieracium intybaceum All.	Switzerlan	Vals	46,61619	10,19876	[86]	2n=2x=18	
Hieracium intybaceum All.	Switzerlan	Grimselpass (Passo del Grimsel)	46,56131	8,33737	[86]	2n=2x=18	
Hieracium intybaceum All.	Italy	Alpe Devero	46,31133	8,25978	[86]	2n=2x=18	
Hieracium intybaceum All.	Italy	Chaz Dura, La Thuile	45,69255	6,92116	[86]	2n=2x=18	
Hieracium intybaceum All.	Austria	Salzburg	47,08135	12,49216	[84]	2n=2x=18 4 plants	Cytometry
Hieracium intybaceum All.	Austria	Steiermark	47,08135	14,57701	[84]	2n=2x=18 3 plants	Cytometry
Hieracium intybaceum All.	Austria	Salzburg	47,25886	12,10541	[84]	2n=2x=18 3 plants	Cytometry
Hieracium intybaceum All.	Italy	Südtirol	46,83766	11,32102	[84]	2n=2x=18 3 plants	Cytometry
Hieracium intybaceum All.	Italy	Südtirol	46,68147	11,71238	[84]	2n=2x=18 3 plants	Cytometry
Hieracium intybaceum All.	Austria	Steiermark	46,91433	13,85483	[84]	2n=2x=18 3 plants	Cytometry
Hieracium intybaceum All.	Italy	Lombardy	46,54238	10,42288	[84]	2n=2x=18 3 plants	Cytometry
Hieracium intybaceum All.	Austria	Kärnten	46,69356	13,92116	[84]	2n=2x=18 5 plants	Cytometry

Hieracium intybaceum All.	Italy	Lombardy	46,53275	10,47719	[84]	2n=2x=18	4 plants	Cytometry
Hieracium intybaceum All.	Austria	Kärnten	46,90493	13,33896	[84]	2n=2x=18	4 plants	Cytometry
Hieracium intybaceum All.	Switzerlan	Ticino	46,47875	8,58911	[84]	2n=2x=18	4 plants	Cytometry
Hieracium intybaceum All.	Austria	Kärnten	46,59670	13,11836	[84]	2n=2x=18	4 plants	Cytometry
Hieracium intybaceum All.	Switzerlan	Ticino	46,53641	8,56783	[84]	2n=2x=18	5 plants	Cytometry
Hieracium intybaceum All.	Austria	Kärnten	46,63178	12,93548	[84]	2n=2x=18	5 plants	Cytometry
Hieracium intybaceum All.	Austria	Ost-tirol	46,88083	12,77623	[84]	2n=2x=18	5 plants	Cytometry
Hieracium intybaceum All.	Switzerlan	Uri	46,65644	8,64261	[84]	2n=2x=18	4 plants	Cytometry
Hieracium intybaceum All.	Italy	Trento	46,88998	12,19655	[84]	2n=2x=18	6 plants	Cytometry
Hieracium intybaceum All.	Switzerlan	Uri	46,57269	8,39544	[84]	2n=2x=18	2 plants	Cytometry
Hieracium intybaceum All.	Italy	Trento	46,81848	11,43463	[84]	2n=2x=18	5 plants	Cytometry
Hieracium intybaceum All.	Switzerlan	Valais	46,37469	7,97711	[84]	2n=2x=18	4 plants	Cytometry
Hieracium intybaceum All.	Italy	Trento	46,73281	10,81566	[84]	2n=2x=18	5 plants	Cytometry
Hieracium intybaceum All.	Italy	Trento	46,88543	11,11846	[84]	2n=2x=18	5 plants	Cytometry
Hieracium intybaceum All.	Austria	Tirol	46,92666	10,17694	[84]	2n=2x=18	4 plants	Cytometry
Hieracium intybaceum All.	Austria	Tirol	46,87850	10,93218	[84]	2n=2x=18	3 plants	Cytometry
Hieracium intybaceum All.	Austria	Tirol	47,01977	12,69444	[84]	2n=2x=18	5 plants	Cytometry
Hieracium intybaceum All.	Austria	Tirol	46,99833	12,59833	[84]	2n=2x=18	6 plants	Cytometry
Hieracium intybaceum All.	Austria	Tirol	47,15611	11,56340	[84]	2n=2x=18	5 plants	Cytometry
Hieracium intybaceum All.	Italy	Valle d'Aosta	45,68008	6,88533	[84]	2n=2x=18	6 plants	Cytometry
Hieracium intybaceum All.	Austria	Tirol	46,86052	11,02427	[84]	2n=2x=18	4 plants	Cytometry
Hieracium intybaceum All.	France	Savoy	45,66275	6,87047	[84]	2n=2x=18	5 plants	Cytometry
Hieracium intybaceum All.	Austria	Tirol	46,47019	11,02427	[84]	2n=2x=18	4 plants	Cytometry
Hieracium intybaceum All.	France	Savoy	45,68552	6,69613	[84]	2n=2x=18	4 plants	Cytometry
Hieracium intybaceum All.	Switzerlan	Graubünden	46,47019	9,75747	[84]	2n=2x=18	5 plants	Cytometry
Hieracium intybaceum All.	Switzerlan	Graubünden	46,74436	9,98238	[84]	2n=2x=18	4 plants	Cytometry
Hieracium intybaceum All.	France	Isére	45,38766	6,13540	[84]	2n=2x=18	5 plants	Cytometry
Hieracium intybaceum All.	France	Savoie	45,28930	6,58873	[84]	2n=2x=18	2 plants	Cytometry
Hieracium intybaceum All.	Austria	Tirol	47,13133	10,20997	[84]	2n=2x=18		Cytometry
Hieracium intybaceum All.	France	Haute-Savoie	45,94933	6,85063	[84]	2n=2x=18		Cytometry
Hieracium jaubertianum Timb.-Lagr. & Lore	France	Languedoc* (Croix de Pelisse?)	43,65351	3,67794	[88]	2n=2x=18	Syn. *H. glaucinum* Jord. subsp. *jaubertianum* (Timb.-Lagr. & Loret)O.Bolòs & Vigo	
Hieracium kittaniae Vladimir.	Bulgaria	Trigrad gorge	41,61515	24,37974	[16]	2n=2x=18		
Hieracium kittaniae Vladimir.	Bulgaria	Devil's Throat Cave (Пещера Дяв	41,61535	24,37957	[35]	2n=2x=18		
Hieracium kittaniae Vladimir.	Bulgaria	Devil's Throat Cave (Пещера Дяв	41,61535	24,37957	[43]	2n=2x=18		
Hieracium kittaniae Vladimir.	Bulgaria	Yagodinska Cave	41,62868	24,32955	[43]	2n=2x=18		
Hieracium korshinskyi Zahn	-	-	-	-	[141]	2n=2x=18	species not included (outside europe), little info	
Hieracium x krasani Woł.	Ukraine	Mt. Hoverla	48,14318	24,48237	[10]	2n=2x=18	*H. transylvanicum* x *H . alpinum* included in *H . alpinum*	
Hieracium x krasani Woł.	Romania	Rodnei Mts., Pietrosul	47,65130	24,64979	[10]	2n=2x=18	*H. transylvanicum* x *H . alpinum* included in *H . alpinum*	
Hieracium laniferum Cav.	Spain	Barranc de Regatxol	40,72457	0,31082	[45]	2n=2x=18		
Hieracium laniferum Cav.	Spain	Barranc de El Salt	40,71086	0,20185	[45]	2n=2x=18		
Hieracium laniferum Cav.	Spain	Barranc de La Fou	40,72485	0,24395	[45]	2n=2x=18		
Hieracium laniferum Cav.	Spain	Ulldecona dam	40,67302	0,23111	[45]	2n=2x=18		
Hieracium laniferum Cav.	Spain	Panta d'ulldecona, Sénia (SW Tor	40,66911	0,24865	[41]	2n=2x=18	glabrous form + hairy-woolly on both leaf sides [Kahle form + form mit beidseitig wollig behaarten blattern]	
Hieracium lawsonii Vill. s.l.	Spain	Organyà (Pont penjant c.)	42,21144	1,33380	[91]	2n=2x=18	sub *H . flocciferum* Arv.-Touv. (see *Hieracium flocculiferum* Zahn subsp. *subflocciferum* Zahn)	
Hieracium lawsonii Vill. s.l.	Spain	Santuario de Nuestra Seniora del	42,25929	2,70714	[91]	2n=2x=18	sub *H . rupicaprinum* Arv.-Touv. & Gaut.*! *Hieracium vellereum* Scheele ex Willk. subsp. *rupicaprinum* (Arv.-Touv. & Gaut.) Mateo, Egido & Gómiz ?	
Hieracium lawsonii Vill. s.l.	Spain	Santuario de Nuestra Seniora del	42,25929	2,70714	[91]	2n=2x=18	*sub H . cordifolium* subsp. *neocerinthe* = *H . neocerinthe* Fr.!	
Hieracium lawsonii Vill. s.l.	Spain	Castellar de n'Hug (34 km Ripoll-I	42,29570	2,02928	[91]	2n=2x=18	*sub H . cordifolium* subsp. *neocerinthe* = *H . neocerinthe* Fr.!	
Hieracium lawsonii Vill. s.l.	Spain	Ermita de Queralt, Berga	42,11019	1,82688	[45]	2n=2x=18	sub. *H . cordifolium* Lapeyr.	
Hieracium lawsonii Vill. s.l.	Andorra	Torrent dels Llimois Valley, Bissis	42,49167	1,44622	[16]	2n=2x=18	sub. *H . cordifolium* Lapeyr.	
Hieracium lawsonii Vill. s.l.	Andorra	Torrent dels Llimois Valley, Bissis	42,49250	1,44555	[35]	2n=2x=18	see Chrtek et al. 2007 (same counts)	
Hieracium legrandianum Arv.-Touv.	Spain	Coll de Josa	42,25043	1,65059	[45]	2n=2x=18	sub *H . amplexicaule* L. icluded in *H . legrandianum*	
Hieracium lucidum Guss.	Italy	Mt. Gallo	38,21675	13,31666	[41]	2n=2x=18		
Hieracium lucidum Guss.	Italy	Mt. Cofano	38,10661	12,66902	[41]	2n=2x=18	*H . lucidum* subsp. *cophanense* (sub *H* . cophanenese Lojac.)	
Hieracium lucidum Guss.	Italy	Mt. Gallo	38,21675	13,31666	[96]	2n=2x=18		
Hieracium lucidum Guss.	Italy	Mt. Cofano	38,10661	12,66902	[97]	2n=2x=18	*H . lucidum* subsp. *cophanense* (sub *H* . cophanenese Lojac.)	
Hieracium lucidum Guss.	Italy	Mt. Passo del Lupo	38,12157	12,75804	[97]	2n=2x=18	*H . lucidum* subsp. *cophanense* (sub *H* . cophanenese Lojac.)	
Hieracium lucidum Guss.	Italy	Mt. Gallo	38,21675	13,31666	[97]	2n=2x=18		
Hieracium lucidum Guss.	Italy	Mt. Passo del Lupo	38,12157	12,75804	[98]	2n=2x=18	*H . lucidum* subsp. *cophanense* (sub *H* . cophanenese Lojac.)	
Hieracium lucidum Guss.	Italy	Mt. Gallo	38,20860	13,29080	[21]	2n=2x=18		
Hieracium lucidum Guss.	Italy	Mt. Passo del Lupo	38,12157	12,75804	[21]	2n=2x=18	*H . lucidum* subsp. *cophanense* (sub *H* . cophanenese Lojac.)	
Hieracium naegelianum Pančić	Macedonia	Mt. Ljuboten	42,20673	21,11936	[81]	2n=2x=18	syn *H . renatae*	
Hieracium naegelianum Pančić	Macedonia	Mt. Sila Vort-Mt. Nistrovski Korab	41,78476	20,61061	[81]	2n=2x=18	syn *H . renatae*	
Hieracium naegelianum Pančić	Macedonia	Mt. Ljuboten	42,20673	21,11936	[81]	2n=2x=18	! subsp. *ljubotenicum* Behr & Zahn	
Hieracium narymense Schischk. & Serg.	-	-	-	-	[142]	2n=2x=18	*Hieracium laevigatum* Willd. ssp. *narymense* (Schischk. & Serg.)?	
Hieracium neocerinthe Fr.	Spain	Santuario de Nuestra Seniora del	42,25929	2,70714	[91]	2n=2x=18	*H . cordifolium* subsp. *neocerinthe* = *H . neocerinthe* Fr.!	
Hieracium neocerinthe Fr.	Spain	Castellar de n'Hug (34 km Ripoll-I	42,29570	2,02928	[91]	2n=2x=18	*H . cordifolium* subsp. *neocerinthe* = *H . neocerinthe* Fr.!	
Hieracium neocerinthe Fr.	Spain	Ermita de Queralt, Berga	42,11019	1,82688	[45]	2n=2x=18	sub. *H . cordifolium* Lapeyr.	
Hieracium neocerinthe Fr.	Andorra	Torrent dels Llimois Valley, Bissis	42,49167	1,44622	[16]	2n=2x=18	sub. *H . cordifolium* Lapeyr.	
Hieracium neocerinthe Fr.	Andorra	Torrent dels Llimois Valley, Bissis	42,49250	1,44555	[35]	2n=2x=18		
Hieracium petrovae Vladimir. & Szeląg	Bulgaria	Buynovo gorge	41,63505	24,33505	[44]	2n=2x=18		
Hieracium petrovae Vladimir. & Szeląg	Bulgaria	Zabrdo	41,78213	24,59572	[44]	2n=2x=18		
Hieracium petrovae Vladimir. & Szeląg	Bulgaria	Trigrad Gorge	41,61512	24,37950	[44]	2n=2x=18	locus calssicus	
Hieracium petrovae Vladimir. & Szeląg	Bulgaria	Trigrad gorge	41,61515	24,37974	[16]	2n=2x=18	locus calssicus	
Hieracium petrovae Vladimir. & Szeląg	Bulgaria	Trigrad gorge	41,66527	24,36388	[35]	2n=2x=18	locus calssicus	
Hieracium petrovae Vladimir. & Szeląg	Bulgaria	Rhodopes Mts. *	41,60292	24,57262	[102]	2n=2x=18	sub. *H . pannosum* group **H . petrovae* ! (only diploid species in sect. Pannosa)	
Hieracium plumulosum A. Kern.	Montenegr	Sinjajevina Mts.	43,12820	19,33110	[46]	2n=2x=18	! Syn. *H . waldsteinii* subsp. *plumulosum*	
Hieracium plumulosum A. Kern.	Montenegr	Mrtvica canyon	42,75296	19,31093	[16]	2n=2x=18	! Syn. *H . waldsteinii* subsp. *plumulosum*	
Hieracium plumulosum A. Kern.	Montenegr	Mrtvica canyon	42,47777	19,81638	[35]	2n=2x=18	?	
Hieracium waldsteinii Tausch	Montenegr	Mojkovac 20 km NWW	43,01427	19,40785	[91]	2n=2x=18	! subsp. *suborieni* Zahn	
Hieracium pojoritense Woł.	Romania	Pojorâta	47,53400	25,49500	[21]	2n=2x=18		
Hieracium pojoritense Woł.	Romania	Adam și Eva hills	47,52133	25,47911	[105]	2n=2x=18	!	
Hieracium pojoritense Woł.	Romania	Slatioara forest, Cheile Latoace ((	47,44428	25,63577	[105]	2n=2x=18	!	
Hieracium pojoritense Woł.	Romania	Pietrele Arse, Valea Caselor (Pârt	47,50761	25,62429	[105]	2n=2x=18	!	
Hieracium pojoritense Woł.	Romania	Bicaz Gorges (Cheile Bicazului)	46,81176	25,82251	[105]	2n=2x=18	!	
Hieracium pojoritense Woł.	Romania	Tulgheș (Mt. Piatra Runcului)?	46,95125	25,76695	[26]	2n=2x=18	generic locality	

Hieracium porrifolium L.	Austria	Karawanken Mts.	46,45197	14,57236	[35]	2n=2x=18	
Hieracium porrifolium L.	Austria	Karawanken Mts.	46,46000	14,50138	[35]	2n=2x=18	
Hieracium porrifolium L.	Slovenia	Julijske Alpe Mts.	46,27555	13,56000	[35]	2n=2x=18	
Hieracium porrifolium L.	Italy	Villini dell'Alpe-Pianizza di Sopra	46,41772	11,21044	[16]	2n=2x=18	
Hieracium porrifolium L.	Austria	Eisenkappel-Vellach-Bad Vellach	46,45630	14,58082	[16]	2n=2x=18	
Hieracium porrifolium L.	Slovenia	Trnovo ob Soci/Ternova d'Isonzo	46,27800	13,55564	[16]	2n=2x=18	
Hieracium porrifolium L.	Slovenia	Mt. Goličica [abstieg nach Mihov	46,43339	13,76962	[76]	2n=2x=18	erroneous coordinates in the article..
Hieracium porrifolium L.	Italy	Cison di Valmarino	45,95779	12,25555	[107]	2n=2x=18	!
Hieracium porrifolium L.	Slovenia	Podljubelj	46,42550	14,26940	[21]	2n=2x=18	
Hieracium porrifolium L.	Slovenia	Julijske Alpe Mts. (Julian Alps) g	46,35720	13,74624	[106]	2n=2x=18	much generic location
Hieracium prenanthoides Vill.	Italy	Claviere	44,94640	6,76920	[21]	2n=2x=18	
Hieracium prenanthoides Vill.	France	Ailefroide	44,90500	6,44140	[21]	2n=2x=18	
Hieracium prenanthoides Vill.	France	Fortresse Marie Thérèse	45,20880	6,73630	[21]	2n=2x=18	
Hieracium prenanthoides Vill.	France	La Grave	45,04361	6,30583	[35]	2n=2x=18	
Hieracium prenanthoides Vill.	France	Montegenèvre	44,92555	6,69694	[35]	2n=2x=18	
Hieracium prenanthoides Vill.	France	Ceillac	44,66063	6,78210	[110]	2n=2x=18	
Hieracium pseudocorymbosum Gremli	Germany	Ostufer des Witznauer Beckens (	47,68897	8,25199	[77]	2n=2x=18	subsp. *petryanum* Zahn
Hieracium racemosum Waldst. & Kit.	Austria	Hall, Tirol (700 m)	47,27159	11,51513	[77]	2n=2x=18	subsp. *leiopsis* Murr & Zahn see *H . leiobium* J.-M.Tison (syn. *H . leiopsis* (Murr & Zahn) Prain)?
Hieracium racemosum Waldst. & Kit.	Italy	M.te Amiata	42,88333	11,61667	[59]	2n=2x=18	
Hieracium racemosum Waldst. & Kit.	-	-	-	-	[41]	2n=2x=18	cultivated
Hieracium recoderi de Retz	Spain	Santuari de Queralt, Berga	42,10768	1,82940	[35]	2n=2x=18	locus classicus
Hieracium recoderi de Retz	Spain	Santuari de Queralt, Berga	42,10768	1,82940	[16]	2n=2x=18	! locus classicus
Hieracium renatae Szeląg	Macedoni	Mt. Solunska Glava (NW slope)	41,70847	21,40863	[47]	2n=2x=18	! locus classicus
Hieracium renatae Szeląg	Macedoni	Gorno Begovo Plateau	41,72736	21,40561	[47]	2n=2x=18	! locus classicus
Hieracium renatae Szeląg	Macedoni	Nezilovska Stena cliff	41,71452	21,41238	[47]	2n=2x=18	! locus classicus
Hieracium renatae Szeląg	Macedoni	Mt. Solunska Glava-Mt. Przal	41,71863	21,41722	[47]	2n=2x=18	! locus classicus
Hieracium renatae Szeląg	Macedoni	Mt. Solunska Glava-Grob pass	41,69119	21,40783	[47]	2n=2x=18	! locus classicus
Hieracium renatae Szeląg	Macedoni	Mt. Solunska Glava	41,70442	21,40517	[81]	2n=2x=18	locus classicus
Hieracium renatae Szeląg	Macedoni	Gorno Begovo Plateau	41,73320	21,41,611	[81]	2n=2x=18	locus classicus
Hieracium renatae Szeląg	Macedoni	Mt. Solunska Glava-Grob pass (2	41,72607	21,39875	[81]	2n=2x=18	locus classicus
Hieracium renatae Szeląg	Macedoni	Mt. Solunska Glava-Mt. Przal (23	41,68667	21,37778	[81]	2n=2x=18	locus classicus
Hieracium renatae Szeląg	Macedoni	Mt. Solunska Glava (2520 m)	41,70308	21,40423	[81]	2n=2x=18	locus classicus
Hieracium sparsum Friv.	Bulgaria	Sestrimo (Сестримо) - Belmeken	42,19875	23,85010	[49]	2n=2x=18	
Hieracium sparsum Friv.	Bulgaria	Maljovica	46,16030	23,38190	[21]	2n=2x=18	
Hieracium sparsum Friv.	Macedoni	Mt. Redir	41,00000	21,77444	[81]	2n=2x=18	
Hieracium sparsum Friv.	Bulgaria	Mt. Vitoša	42,56861	23,29888	[35]	2n=2x=18	
Hieracium sparsum Friv.	Bulgaria	Pirin Mts. (Bezbozko (Bezbog) la	41,73833	23,52527	[35]	2n=2x=18	
Hieracium sparsum Friv.	Serbia	Mt. Besna Kobila	42,53071	22,23442	[46]	2n=2x=18	
Hieracium sparsum Friv.	Bulgaria	Mt. Mali Ruen (Връх Мали Руен)	42,16508	22,52797	[113]	2n=2x=18	
Hieracium sparsum Friv.	Bulgaria	Demyanitsa (Demyanitsa Hut)	41,74430	23,46721	[113]	2n=2x=18	
Hieracium sparsum Friv.	Bulgaria	Mt. Vihren	41,76079	23,40936	[113]	2n=2x=18	
Hieracium sparsum Friv.	Bulgaria	Mt. Rila	42,13377	23,54961	[103]	2n=2x=18	
Hieracium sparsum Friv.	-	-	-	-	[112]	2n=2x=18	on plant of unknown origin
Hieracium sparsum Friv.	Bulgaria	Mt. Goliam Perelik	41,60673	24,57424	[48]	2n=2x=18	sub. Hieracium cernuum Friv.*
Hieracium sparsum Friv.	Macedoni	Nidze Mts.	40,98010	21,80375	[81]	2n=2x=18	sub. Hieracium cernuum Friv.*
Hieracium speciosum Hornem.	-	-	-	-	[25]	2n=2x=18	Lund Botanic Garden (unknown origin)
Hieracium stelligerum Froel.	France	Le Pont d'Arc	44,38234	4,41651	[35]	2n=2x=18	
Hieracium stelligerum Froel.	France	Le Pont d'Arc	44,38234	4,41651	[16]	2n=2x=18	
Hieracium stelligerum Froel.	France	Jaujac	44,64000	4,25400	[21]	2n=2x=18	
Hieracium stelligerum Froel.	France	Jaujac	44,64000	4,25400	[21]	2n=2x=18	
Hieracium stelligerum Froel.	France	Thueyts	44,67080	4,21930	[21]	2n=2x=18	
Hieracium stelligerum Froel.	France	Thueyts	44,67000	4,21000	[21]	2n=2x=18	
Hieracium stelligerum Froel.	France	Thueyts	44,67000	4,21060	[21]	2n=2x=18	
Hieracium tomentosum L.	France	Col de Tende	43,13878	7,56583	[35]	2n=2x=18	
Hieracium tomentosum L.	France	Col de Tende	44,13878	7,56583	[16]	2n=2x=18	
Hieracium transylavanicum Heuff.	Romania	Hargita Mts.	46,14863	25,84941	[69]	2n=2x=18	
Hieracium transylavanicum Heuff.	Romania	Apuseni Mts.	46,62697	22,66186	[69]	2n=2x=18	
Hieracium transylavanicum Heuff.	Romania	Hagimas Mts.	46,66921	25,83316	[69]	2n=2x=18	
Hieracium transylavanicum Heuff.	Ukraine	Mt. Hoverla	48,14335	24,48239	[10]	2n=2x=18	
Hieracium transylavanicum Heuff.	Romania	Rodnei Mts., Pietrosul	47,65130	24,64979	[10]	2n=2x=18	
Hieracium transylavanicum Heuff.	Monteneg	Durmitor Mts.	43,14986	19,09443	[46]	2n=2x=18	
Hieracium transylavanicum Heuff.	Bosnia He	Prokoško jezero lake	43,96023	17,75520	[48]	2n=2x=18	
Hieracium transylavanicum Heuff.	Bosnia He	Dragaš pass	41,96324	20,64943	[48]	2n=2x=18	
Hieracium transylavanicum Heuff.	Croatia	Rude	45,77867	15,68340	[48]	2n=2x=18	
Hieracium transylavanicum Heuff.	Romania	Valea Crăpăturii Gorge	45,56609	25,26436	[48]	2n=2x=18	
Hieracium transylavanicum Heuff.	Slovenia	Mt. Donačka gora	46,26336	15,74196	[48]	2n=2x=18	
Hieracium transylavanicum Heuff.	Ukraine	Mt. Berlebashka	47,93694	24,35861	[35]	2n=2x=18	
Hieracium transylavanicum Heuff.	Ukraine	Mt. Berlebashka	47,96187	24,30322	[16]	2n=2x=18	
Hieracium transylavanicum Heuff.	Romania	Mt. Pietrosul Bogolin	47,38770	25,53630	[21]	2n=2x=18	
Hieracium transylavanicum Heuff.	Romania	Băile Tuşnad	46,14700	25,85100	[21]	2n=2x=18	
Hieracium transylavanicum Heuff.	Ukraine	Chornohora Mts.	48,16104	24,50047	[114]	2n=2x=18	
Hieracium transylavanicum Heuff.	Bulgaria	Kopren mountain hut (хижа Копр	43,32754	22,86129	[51]	2n=2x=18	
Hieracium transylavanicum Heuff.	Monteneg	Durmitor Mts. Crno jezero Lake	43,14975	19,09443	[58]	2n=2x=18	
Hieracium transylavanicum Heuff.	Monteneg	Bjelasica Mts. Biogradsko jezero	42,89870	19,60720	[58]	2n=2x=18	
Hieracium transylavanicum Heuff.	Serbia	Tara Mts. Mitrovac	43,91386	19,42440	[58]	2n=2x=18	
Hieracium transylavanicum Heuff.	Bulgaria	Chiprovska Mts. Mt. Kopren	43,33563	22,86386	[58]	2n=2x=18	
Hieracium transylavanicum Heuff.	Romania	Mehedinti Mts. Mt. Domugled	44,88194	22,43972	[58]	2n=2x=18	
Hieracium transylavanicum Heuff.	Romania	Muntele Mare Mts. Mt. Buscat	46,53610	23,16890	[58]	2n=2x=18	
Hieracium transylavanicum Heuff.	Romania	Hargita Mts. Mt. Ciceu	46,39237	25,63543	[58]	2n=2x=18	
Hieracium transylavanicum Heuff.	Romania	Ceahlău Mts. Mt. Toaca	46,99583	25,92082	[58]	2n=2x=18	
Hieracium transylavanicum Heuff.	-	-	-	-	[24]	2n=2x=18	Garden plant > no locality
Hieracium transylavanicum Heuff.	Ukraine	Luhy-Mt. Goverla (Hoverla)	58,07390	24,44826	[42]	2n=2x=18	
Hieracium transylavanicum Heuff.	Ukraine	Kuzyi - Trybushany (4km W Dilov	47,93929	24,14051	[42]	2n=2x=18	
Hieracium transylavanicum Heuff.	Romania	Mt. Pietrosul Mare	47,65000	24,65000	[26]	2n=2x=18	
Hieracium transylavanicum Heuff.	Monteneg	Durmitor Mts, Crno jezero Lake	43,14972	19,09443	[58]	2n=2x=18	

Hieracium transylavanicum Heuff.	Monteneg	Bjelasica Mts, Biogradsko jezero	42,89863	19,60722	[58]	2n=2x=18		
Hieracium transylavanicum Heuff.	Serbia	Tara Mts, Mitrovac	43,91930	19,42442	[58]	2n=2x=18		
Hieracium transylavanicum Heuff.	Bulgaria	Western Stara Planina Mts, Chipr	43,33556	22,86388	[58]	2n=2x=18		
Hieracium transylavanicum Heuff.	Romania	Mehedinti Mts, Mt. Domugled	44,88194	22,43973	[58]	2n=2x=18		
Hieracium transylavanicum Heuff.	Romania	Apuseni Mts, Muntele Mare Mts,	46,53612	23,16888	[58]	2n=2x=18		
Hieracium transylavanicum Heuff.	Romania	Hargita Mts, Mt. Ciceu	46,39250	25,93556	[58]	2n=2x=18		
Hieracium transylavanicum Heuff.	Romania	Ceahlău Mts, Mt. Toaca	46,99583	25,92082	[58]	2n=2x=18		
Hieracium transylavanicum Heuff.	-	-	-	-	[24]	2n=2x=18	cultivated sub *H . rotundatum* Kit. ex Schult.	
Hieracium transylavanicum Heuff.	Bulgaria	Western Stara Planina Mt.*	43,26368	23,00715	[102]	2n=2x=18	*generic location! sub *H . rotundatum* Kit. ex Schult.	
Hieracium umbellatum L.	Poland	Jantar	54,33333	19,040483	[35]	2n=2x=18		
Hieracium umbellatum L.	Czech Rep	Praha-Troja	50,12166	14,40388	[35]	2n=2x=18		
Hieracium umbellatum L.	Germany	Hörnum, Island of Sylt	54,75138	8,28555	[35]	2n=2x=18		
Hieracium umbellatum L.	Czech Rep	Pusta vinice', Praha-Troja	50,12178	14,40389	[16]	2n=2x=18		
Hieracium umbellatum L.	Ukraine	Mt. Pikui	48,83127	22,99583	[16]	2n=2x=18		
Hieracium umbellatum L.	Slovakia	Prakovce zastávka	48,81680	20,89416	[15]	2n=2x=18		
Hieracium umbellatum L.	Slovakia	Prakovce (Volovské vrchy Mts.)	48,81500	20,91477	[26]	2n=2x=18		
Hieracium umbellatum L.	Austria	Axams	47,24781	11,27678	[77]	2n=2x=18	subsp. *brevifolioides* Zahn	
Hieracium umbellatum L.	Germany	Altomünster	48,35393	11,22452	[77]	2n=2x=18		
Hieracium umbellatum L.	Germany	Burgruine Kallmünz	49,16357	11,95185	[77]	2n=2x=18		
Hieracium umbellatum L.	Poland	Mogilany	49,93335	11,86671	[132]	2n=2x=18		
Hieracium umbellatum L.	Finland	Kuivaniemi (Church)	65,57894	25,18767	[130]	2n=2x=18		
Hieracium umbellatum L.	Finland	Valikangas (Pohjanmaa)	63,52951	24,08800	[130]	2n=2x=18	sub *H . laevigatum* Willd./ aggr. *H . umbellatum* L.	
Hieracium umbellatum L.	Sweden	Skellefteå	64,71940	21,01220	[21]	2n=2x=18		
Hieracium umbellatum L.	Sweden	Gävle	60,69110	17,08390	[21]	2n=2x=18		
Hieracium umbellatum L.	Cechia	Velké Žernoseky	50,54620	14,05360	[21]	2n=2x=18		
Hieracium umbellatum L.	Cechia	Libochovany	50,54660	14,04990	[21]	2n=2x=18		
Hieracium umbellatum L.	Slovakia	Prakovce	48,81400	20,91200	[21]	2n=2x=18		
Hieracium umbellatum L.	Slovakia	Prakovce	48,81500	20,91200	[21]	2n=2x=18		
Hieracium umbellatum L.	Slovakia	Hlohovec, Urbánok hill	48,41714	17,78949	[123]	2n=2x=18		
Hieracium umbellatum L.	Norway	Snåsavatnet	64,26250	12,27060	[21]	2n=2x=18		
Hieracium umbellatum L.	Cechia	Libochovany	50,55590	14,04040	[21]	2n=2x=18		
Hieracium umbellatum L.	France	Col du Lautaret	45,03810	6,404240	[21]	2n=2x=18		
Hieracium umbellatum L.	Bulgaria	Western Balkan Foothills region*	43,02222	23,14620	[102]	2n=2x=18	*generic locality	
Hieracium umbellatum L.	Greece	Mt. Olympus, N foothills	40,18116	22,34029	[128]	2n=2x=18		
Hieracium umbellatum L.	Germany	Macklengur, Darss (Amt Darß/Fis	54,23912	12,24945	[116]	2n=2x=18		
Hieracium umbellatum L.	Germany	Greifswald	54,11358	13,43837	[116]	2n=2x=18		
Hieracium umbellatum L.	Sweden	Vitemölla Beach	55,71061	14,19950	[117]	2n=2x=18	3-4 conunts	
Hieracium umbellatum L.	Sweden	Hagestad	55,38681	14,14612	[117]	2n=2x=18		
Hieracium umbellatum L.	Sweden	Löderup	55,40716	14,17355	[117]	2n=2x=18		
Hieracium umbellatum L.	Sweden	Norreborg	55,91378	12,71119	[117]	2n=2x=18		
Hieracium umbellatum L.	Sweden	Hallands Väderö	56,44246	12,56182	[117]	2n=2x=18	2 different counts from the same island	
Hieracium umbellatum L.	Sweden	Osby	56,36554	14,00226	[117]	2n=2x=18		
Hieracium umbellatum L.	Sweden	Vårhallarna (Norra Infartsvägen F	55,57915	14,33176	[117]	2n=2x=18		
Hieracium umbellatum L.	Sweden	Brantevik	55,51165	14,34838	[117]	2n=2x=18		
Hieracium umbellatum L.	Sweden	Maglarp (near the shore)	55,37142	13,07422	[117]	2n=2x=18		
Hieracium umbellatum L.	Sweden	Skåne*	56,02951	13,63715	[117]	2n=2x=18		
Hieracium umbellatum L.	Finland	*	-	-	[118]	2n=2x=18	*generic locality	
Hieracium umbellatum L.	Finland	*	-	-	[119]	2n=2x=18	*generic locality	
Hieracium umbellatum L.	England	King Harry Ferry	50,21692	-5,02881	[126]	2n=2x=18		
Hieracium umbellatum L.	Hungary	Hajdúbagos*	47,40220	21,68956	[115]	2n=2x=18		*generic locality
Hieracium umbellatum L.	Norway	Nøtterøy	59,18008	10,45633	[122]	2n=2x=18		
Hieracium umbellatum L.	Germany	Schleswig-Holstein*	54,27797	8,87416	[116]	2n=2x=18	record in https://chromosomes.senckenberg.de/	*generic locality
Hieracium umbellatum L.	Belarus	Wilejka (Poland? or Belarus!)	54,49938	26,95598	[124]	2n=2x=18		
Hieracium umbellatum L.	Poland	Białowieża forest	52,69761	23,80527	[124]	2n=2x=18		
Hieracium umbellatum L.	Poland	Brzeźno, Gdansk	54,41658	18,61561	[124]	2n=2x=18		
Hieracium umbellatum L.	Poland	Łeba (Wydma Łącka?)	54,74821	17,43230	[124]	2n=2x=18		
Hieracium umbellatum L.	Slovakia	Devínska Kobyla	48,18905	16,99288	[127]	2n=2x=18		
Hieracium umbellatum L.	Slovakia	Devínska Kobyla	48,18905	16,99288	[127]	2n=2x=18	sub. *H . sabaudum* L. (to be inlcuded in *H . umbellatum* ..)	
Hieracium umbellatum L.	Netherlanc	L'Aia (Den Haag)	52,10749	4,31879	[129]	2n=2x=18		
Hieracium umbellatum L.	Sweden	near Stockholm*	59,31430	17,97792	[22]	2n=2x=18	*generic locality	
Hieracium umbellatum L.	Netherlanc	Schiermonnikoog	53,47773	6,15833	[121]	2n=2x=18		
Hieracium umbellatum L.	Netherlanc	Boschplaat, Terschelling	53,42801	5,46705	[120]	2n=2x=18		
Hieracium umbellatum L.	Netherlanc	Hollum, Ameland	53,43723	5,64035	[125]	2n=2x=18		
Hieracium umbellatum L.	Netherlanc	Oostvoorne, Heveringseweg (?)	51,90397	4,09649	[125]	2n=2x=18		
Hieracium umbellatum L.	U.S.A	Lake co., Mont. (Lake County Mo	-	-	[137]	2n=2x=18	sub. *H . canadense* Michx.	
Hieracium umbellatum L.	Slovakia	Považský Inovec	48,59836	17,91865	[131]	2n=2x=18	(just the abstract seen)	
Hieracium umbellatum L.	Turkey	Ayder Yaylası Yolu, Çamlıhemşin,	40,99843	41,05374	[133]	2n=2x=18		
Hieracium umbellatum L.	Turkey	Taşkıran (?)	40,67863	40,25710	[133]	2n=2x=18	generic locality	
Hieracium umbellatum L.	Polonia	Buków	49,95051	19,83333	[132]	2n=2x=18	! Sub *Hieracium laurinum* Arvet-Touvet; Hieracium vasconicum subsp. *laureolum* (Arv.-Touv.) Greuter	
Hieracium umbellatum L.	Ukraine	Mt. Dogyas'ka	48,30000	24,16667	[26]	2n=2x=18	sub Hieracium hryniaviense Woł. (?*Hieracium raddeanum* Zahn subsp. *hryniaviense* (Woł.) Greuter)	
Hieracium umbellatum L.	Ukraine	Mt. Gereseska (Svydovets Mts.*)	48,25689	24,16885	[42]	2n=2x=18	sub H. conicum Arv.-Touv.generic locality	
Hieracium umbellatum L.	Ukraine	Mt. Pip Ivan	48,04557	24,63267	[42]	2n=2x=18	sub H. conicum Arv.-Touv.generic locality	
Hieracium hryniaviense Woł.	Ukraine	Mt. Dogyas'ka	48,30000	24,16667	[26]	2n=2x=18	to be included in H. umbellatum?! *Hieracium raddeanum* Zahn subsp. *hryniaviense* (Woł.) Greuter	
Hieracium valdepilosum Vill.	Austria	W Baad 2000-2050 m	47,29981	10,0768	[76]	2n=2x=18	! subsp. *subsinuatum* (Nägeli & Peter) Zahn	
Hieracium virgaurea Coss.	Italy	Mt. Amiata	42,883333	11,61667	[59]	2n=2x=18	*Hieracium racemosum* Waldst. & Kit. ex Willd. subsp. *virgaurea* (Coss.) Zahn	
Hieracium virgicaule Nägeli & Peter	-	-	-	-	[139]	2n=2x=18	cfr. H. umbellatum! Cited in Mraz & Paule 2006	
Hieracium vranceae Mráz	Romania	Piatra Ciutei	45,94425	26,58862	[33]	2n=2x=18		
Hieracium vranceae Mráz	Romania	Cheile Tișiței	45,94039	26,5852	[33]	2n=2x=18		
Hieracium vranceae Mráz	Bulgaria	Rhodopes Mts.*	-	-	[103]	2n=2x=18	*generic locality ! H. sp. ? sect. Glauciformia (Freyn) Zahn	
